# Echinatin effectively protects against NLRP3 inflammasome–driven diseases by targeting HSP90

**DOI:** 10.1172/jci.insight.134601

**Published:** 2021-01-25

**Authors:** Guang Xu, Shubin Fu, Xiaoyan Zhan, Zhilei Wang, Ping Zhang, Wei Shi, Nan Qin, Yuanyuan Chen, Chunyu Wang, Ming Niu, Yuming Guo, Jiabo Wang, Zhaofang Bai, Xiaohe Xiao

**Affiliations:** 1 Military Institute of Chinese Materia, the Fifth Medical Centre, General Hospital of PLA, Beijing, China.; 2 Integrative Medical Centre, the Fifth Medical Centre, General Hospital of PLA, Beijing, China.; 3 School of Pharmacy, Jiangxi University of Traditional Chinese Medicine, Nanchang, China.; 4 Jiujiang Institute for Food and Drug Control, Jiujiang, China.; 5 School of Pharmacy, Chengdu University of Traditional Chinese Medicine, Chengdu, China.

**Keywords:** Immunology, Inflammation, Innate immunity

## Abstract

Aberrant activation of NLRP3 inflammasome has been implicated in a variety of human inflammatory diseases, but currently, no pharmacological NLRP3 inhibitor has been approved. In this study, we showed that echinatin, the ingredient of the traditional herbal medicine licorice, effectively suppresses the activation of NLRP3 inflammasome in vitro and in vivo. Further investigation revealed that echinatin exerts its inhibitory effect on NLRP3 inflammasome by binding to heat-shock protein 90 (HSP90), inhibiting its ATPase activity and disrupting the association between the cochaperone SGT1 and HSP90-NLRP3. Importantly, in vivo experiments demonstrated that administration of echinatin obviously inhibits NLRP3 inflammasome activation and ameliorates LPS-induced septic shock and dextran sodium sulfate–induced (DSS-induced) colitis in mice. Moreover, echinatin exerted favorable pharmacological effects on liver inflammation and fibrosis in a mouse model of nonalcoholic steatohepatitis (NASH). Collectively, our study identifies echinatin as a potentially novel inhibitor of NLRP3 inflammasome, and its use may be developed as a therapeutic approach for the treatment of NLRP3-driven diseases.

## Introduction

Inflammasome is a large multimolecular platform consisting of apoptosis-associated speck-like protein containing a caspase recruitment domain (CARD; ASC), caspase-1, and innate immune sensors, including NOD-like receptor (NLR) family members and non-NLR receptors (1–4). As one of the most fundamental and the best characterized immune sensors, NLRP3 inflammasome can be activated in response to diverse damage-associated and pathogen-associated molecular patterns (DAMPs and PAMPs, respectively), such as extracellular ATP, monosodium urate crystals (MSU), poly(I:C), nigericin, influenza A virus, *Listeria monocytogenes*, and more (5). Once activated, NLRP3 nucleates the oligomerization of ASC into a helical filament formation, resulting in formation of a single macromolecular focus, the so-called ASC speck (4), which acts as the platform for autocleavage and activation of pro–caspase-1, subsequently leading to pyroptosis and the processing of IL-1β and IL-18. However, the aberrant activation of NLRP3 inflammasome plays a crucial role in the pathogenesis of several human diseases, such as cryopyrin-associated autoinflammatory syndromes (CAPS), type 2 diabetes (T2D), gout, atherosclerosis, and neurodegenerative diseases (6–13). Given the robust inflammatory potential of NLRP3 and its role in pathogenesis, targeting NLRP3 inflammasome has been proven to be an attractive pharmacological approach for treating the inflammatory diseases (5, 14). Although several small-molecule compounds targeting NLRP3 inflammasome have shown therapeutic benefits on NLRP3-driven diseases in a variety of animal models (5, 14–22), none have been used in clinic (14).

Echinatin is a bioactive flavonoid of *Glycyrrhiza* plants (licorice), which is a well-recognized natural sweetening and flavoring food additive and has been extensively used in traditional herbal medicine (19). It has been reported that echinatin displays cardioprotective effects against ischemia/reperfusion injury of the heart by partially inhibiting angiotensin converting enzyme (ACE) (23, 24). In the European patent EP0998939A1, echinatin can effectively inhibit the irritant compound 12-O-tetradecanoylphorbol-13-acetate–induced (TPA-induced) erythema and can show therapeutic properties for psoriasis treatment. Moreover, biological studies have revealed that echinatin possesses antioxidant and antiinflammatory activities (23, 25) and acts as a potent activator of antioxidant transcription factor nuclear erythroid 2-related factor 2 (Nrf2) and attenuates CCl_4_-induced acute liver injury in mice (26, 27). It has been shown that echinatin could also inhibit LPS-induced prostaglandin E2 (PGE2), IL-6, ROS, and nitric oxide (NO) production in vitro (25). Despite the emerging beneficial effects of echinatin, the underlying molecular mechanisms and the direct target remain unclear.

In this study, we show that echinatin could remarkably inhibit the NLRP3 inflammasome activation. Upon binding to HSP90 and suppressing its ATPase activity, and disrupting the interaction between the cochaperone SGT1 and HSP90-NLRP3, echinatin prevents ASC oligomerization and the subsequent inflammasome activation, and it exhibits the remarkable therapeutic effects in several mouse models of NLRP3-dependent inflammatory diseases.

## Results

### Echinatin inhibits NLRP3 inflammasome activation in mouse BMDMs and human PBMCs.

**Figure 1:**
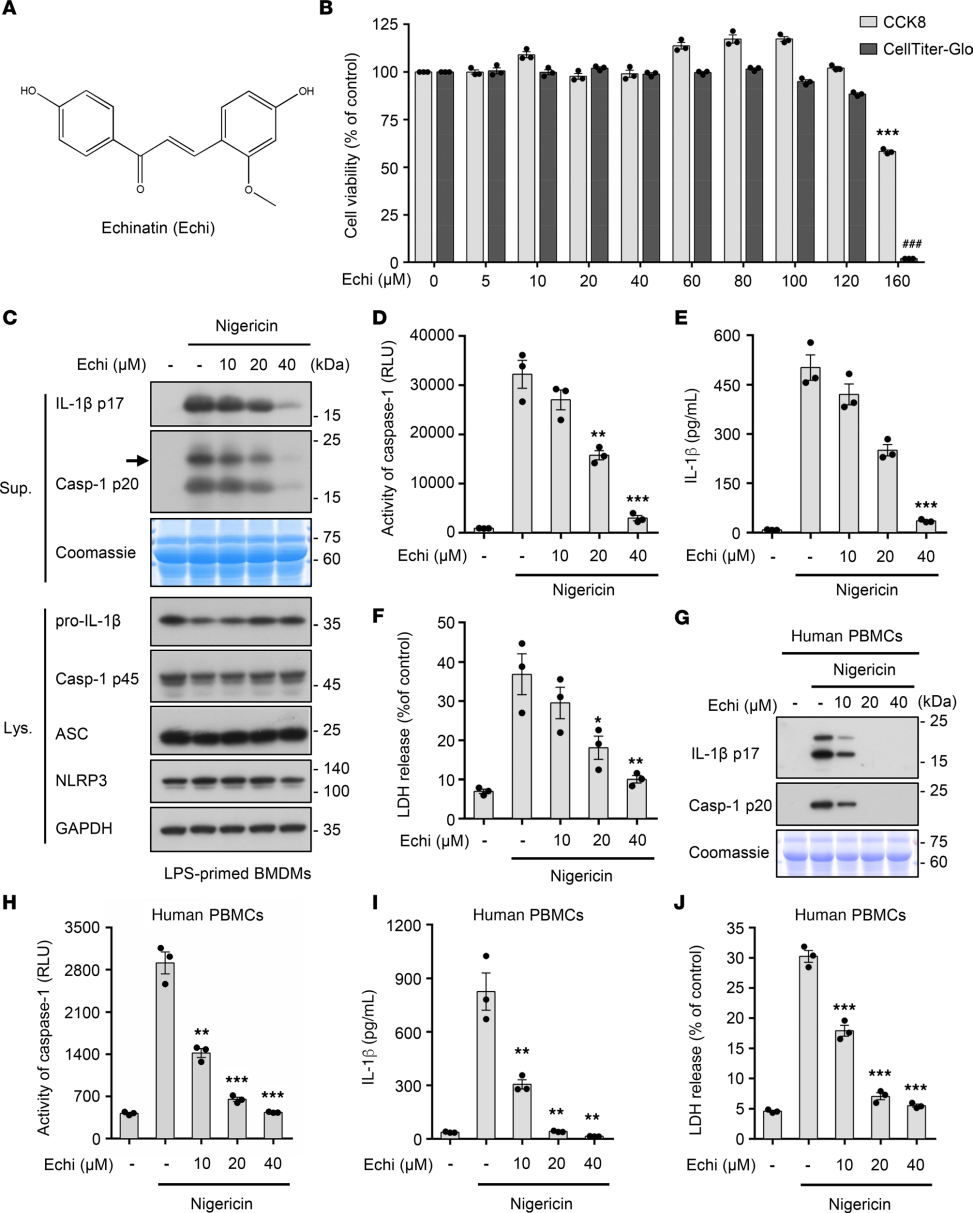
Echinatin inhibits NLRP3 inflammasome activation in mouse BMDMs and human PBMCs. (**A**) The structure of echinatin (Echi) is shown. (**B**) Cell viability of BMDMs treated with an indicated dose of echinatin was assessed using Cell Counting Kit-8 (CCK-8) or CellTiter-Glo Assay, which is based on quantitation of ATP. (**C**–**F**) LPS-primed BMDMs were pretreated with various doses of echinatin and then stimulated with nigericin, cleaved caspase-1 and production of IL-1β (**C**). Activity of caspase-1 (**D**), secretion of IL-1β (**E**), and LDH (**F**) in SN were assessed. (**G**–**J**) LPS-primed human PBMCs were pretreated with various doses of echinatin and then stimulated with nigericin, cleaved caspase-1 and production of IL-1β (**C**). Activity of caspase-1 (**D**), secretion of IL-1β (**E**), and LDH (**F**) in SN were assessed. Data are expressed as mean ± SEM (*n* = 3/group, resulting from 3 independent experiments). One-way ANOVA, followed by Dunnett’s post hoc test, was used to assess the differences of multiple groups (**B**, **D**–**F**, and **H**–**J**). *P < 0.05, **P < 0.01, ***P < 0.001, ^###^*P* < 0.001 compared with control (**B**) or control with stimulated (**D**–**F**, **H**–**J**).

To investigate the effect of echinatin (Figure 1A) on NLRP3 inflammasome activation, we first tested the cytotoxicity of echinatin in BM-derived macrophages (BMDMs). Cell viability assays showed that echinatin did not exhibit any cytotoxicity at the dose below 100 μM in BMDMs (Figure 1B). Next, we evaluated the impact of echinatin on caspase-1 cleavage and IL-1β secretion. BMDMs were first primed with LPS and then incubated with a range of echinatin concentrations for 60 minutes before stimulation with nigericin to induce NLRP3 inflammasome activation. As shown in Figure 1, C–F, echinatin significantly inhibited caspase-1 activation, IL-1β maturation, and LDH release triggered by nigericin in LPS-primed BMDMs. Meanwhile, the production of TNF-α, an inflammasome-independent cytokine, was not affected by echinatin (Supplemental Figure 1A; supplemental material available online with this article; https://doi.org/10.1172/jci.insight.134601DS1). Similarly, ATP-induced NLRP3 inflammasome activation was also blocked by echinatin (Supplemental Figure 2, A–E). In addition, we found that echinatin did not affect the expression of NLRP3 and pro–IL-1β (Figure 1C and Supplemental Figure 2A). Moreover, our data show that nigericin-induced caspase-1 cleavage and IL-1β secretion in LPS-primed human peripheral blood mononuclear cells (hPBMCs) could also be dramatically impaired by echinatin (Figure 1, G–J, and Supplemental Figure 1, B–C), and the inhibitory effect of echinatin on NLRP3 inflammasome activation in hPBMCs was about 2 times more potent than its effect in BMDMs (Figure 1, D, E, H, and I). Taken together, these data indicate that echinatin inhibits nigericin-induced NLRP3 inflammasome activation in both mouse and human immune cells.

### Echinatin suppresses multiple agonist–mediated NLRP3 inflammasome activation and assembly.

**Figure 2:**
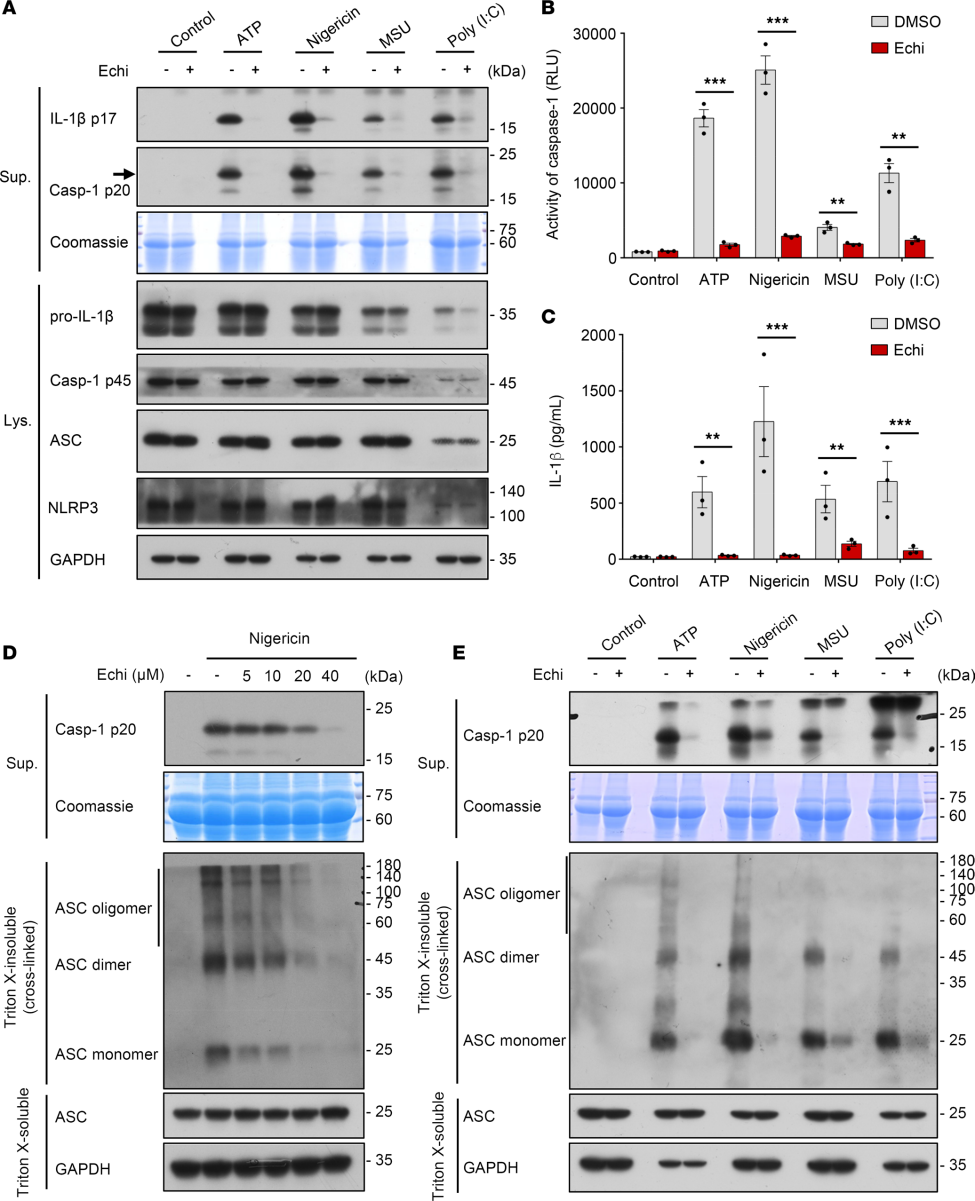
Echinatin suppresses multiple agonist–mediated NLRP3 inflammasome activation and assembly. (**A**–**C**) LPS-primed BMDMs were pretreated with echinatin (40 μM) or vehicle and then stimulated with ATP, nigericin, MSU, and poly(I:C); cleaved caspase-1 and production of IL-1β were examined by IB analysis (**A**); activity of caspase-1 (**B**) and secretion of IL-1β (**C**) in SN were assessed. (**D**) LPS-primed BMDMs were pretreated with indicated dose of echinatin and stimulated with nigericin. IB analysis was used to detect cross-linked ASC in the Triton X-insoluble pellet. (**E**) IB analysis of cross-linked ASC in the Triton X–insoluble pellet from LPS-primed BMDMs pretreated with echinatin (40 μM) or vehicle and then stimulated with ATP, nigericin, MSU, and poly(I:C). Data are expressed as mean ± SEM (*n* = 3/group, resulting from 3 independent experiments). Statistics differences were analyzed by unpaired *t* test (**B** and **C**). **P < 0.01, ***P < 0.001.

**Figure 3:**
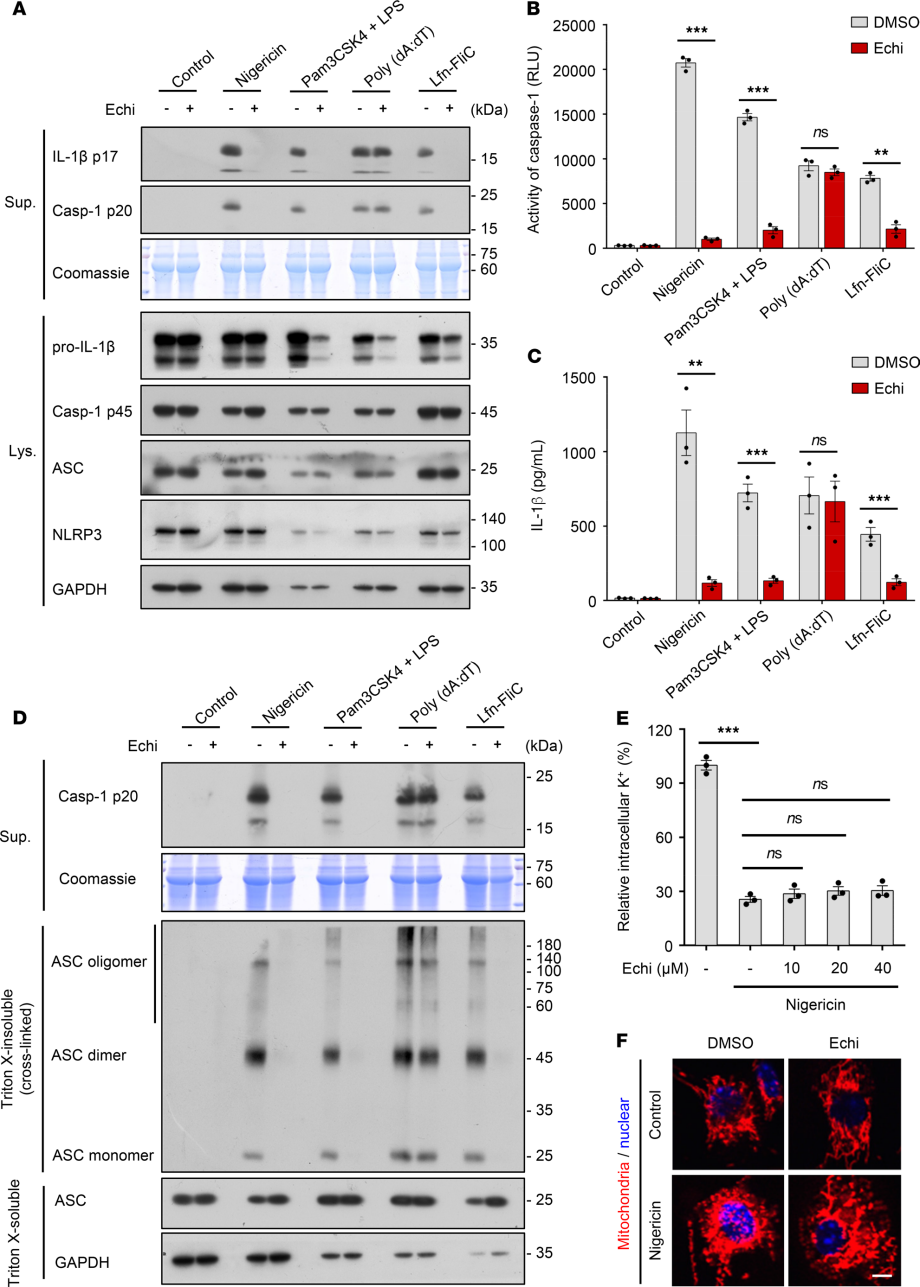
Echinatin does not directly target the ASC oligomerization and does not block K^+^ efflux or mitochondrial damage. (**A**–**C**) LPS-primed BMDMs were pretreated with echinatin (40 μM) or vehicle and then stimulated with nigericin, poly(dA:dT), and Lfn-FliC — or Pam3CSK4-primed BMDMs were pretreated with echinatin (40 μM) or vehicle and then stimulated with transfected LPS. Cleaved caspase-1 and production of IL-1β were examined by IB analysis (**A**), and activity of caspase-1 (**B**) and secretion of IL-1β (**C**) in SN were assessed. (**D**) IB analysis of cross-linked ASC in the Triton X–insoluble pellet from LPS-primed BMDMs pretreated with echinatin (40 μM) or vehicle and then stimulated with nigericin, poly(dA:dT), Lfn-FliC, or Pam3CSK4-primed BMDMs were pretreated with echinatin (40 μM) or vehicle and then stimulated with transfected LPS. (**E**) Qualification of intracellular potassium in LPS-primed BMDMs pretreated with indicated dose of echinatin and stimulated with nigericin. (**F**) Staining with MitoTracker red in LPS-primed BMDMs pretreated with echinatin (40 μM) or vehicle and then stimulated with nigericin. Scale bar: 5 μm. Data are expressed as mean ± SEM (*n* = 3/group, resulting from 3 independent experiments). Statistics differences were analyzed by unpaired *t* test (**B** and **C**) or 1-way ANOVA followed by Dunnett’s post hoc test (**E**). **P < 0.01, ***P < 0.001.

Next, we tested the role of echinatin in the activation of other stimuli-induced NLRP3 inflammasomes. We treated LPS-primed BMDMs with echinatin and then stimulated them with ATP, MSU, or poly(I:C); we found that caspase-1 activation and IL-1β secretion in response to those canonical NLRP3 stimuli were potently suppressed by echinatin (Figure 2, A–C). Intracellular LPS or gram-negative bacteria can activate caspase-11 to initiate noncanonical NLRP3 inflammasome activation (28–31). We then evaluated the effect of echinatin on the noncanonical pathway. The results showed that LPS transfection induced caspase-11–dependent caspase-1 cleavage and IL-1β secretion in Pam3CSK4-primed BMDMs, whereas pretreatment with echinatin before intracellular LPS stimulation resulted in the inhibition of noncanonical NLRP3 inflammasome activation (Figure 3, A–C). Likewise, the production of TNF-α was not affected by echinatin (Supplemental Figure 1, D and E). These results suggest that echinatin is a broad inhibitor of NLRP3 inflammasome, which could inhibit both canonical and noncanonical NLRP3 inflammasome activation.

Then, we investigated the mechanisms of how echinatin suppressed NLRP3 inflammasome activation. ASC oligomerization is a critical step for NLRP3 inflammasome activation (32). After stimulation with nigericin, ASC monomers and higher-order complexes were detected by immunoblot analysis. Consistent with the blocking effects of echinatin on caspase-1 cleavage, echinatin also attenuated nigericin-stimulated ASC oligomerization in a dose-dependent manner (Figure 2D). Further investigation showed that echinatin effectively blocked ASC oligomerization induced by other NLRP3 agonists, such as ATP, MSU, and poly(I:C) (Figure 2E). These results suggest that echinatin may directly act on ASC oligomerization or affect its upstream events during NLRP3 inflammasome assembly.

### Echinatin does not directly target the ASC oligomerization and does not block K^+^ efflux or mitochondrial damage.

ASC oligomerization is indispensable for the activation of NLRP3 and AIM2 inflammasome, but it is dispensable for NLRC4 inflammasome activation (33, 34). Next, we further explored whether the NLRP3 inflammasome inhibition caused by echinatin treatment is due to the direct targeting of ASC oligomerization. We first tested the influence of echinatin on the activation of AIM2 and NLRC4 inflammasomes, which were activated by double-stranded DNA mimic poly(dA:dT) and Lfn-FliC, respectively (35). The results showed that echinatin specifically inhibited Lfn-FliC–induced caspase-1 activation and IL-1β secretion in LPS-primed BMDMs (Figure 3, A–C, and Supplemental Figure 1E), whereas the production of active caspase-1 and mature IL-1β triggered by poly(dA:dT) transfection were not affected (Figure 3, A–C). Those data indicate that echinatin does not regulate AIM2 inflammasome activation. Next, we examined the effect of echinatin on ASC oligomer formation triggered by related stimuli. In accordance with the effect of echinatin on the Lfn-FliC– and poly(dA:dT)–induced caspase-1 processing, echinatin remarkably inhibited ASC oligomerization induced by Lfn-FliC, but not poly(dA:dT) (Figure 3D). These data suggest that echinatin does not directly target ASC oligomerization to inhibit NLRP3 inflammasome activation.

To further elucidate the mechanism of how echinatin affects NLRP3 inflammasome activation, we tested the influence of echinatin on the common upstream trigger for all examined agonist-mediated NLRP3 inflammasome activation, the K^+^ efflux (36, 37). When LPS-primed BMDMs were treated with nigericin, the intracellular potassium dramatically decreased, but the decrease was equally observed upon echinatin treatment (Figure 3E). In addition, mitochondrial damage — represented as mitochondrial fragmentation, oxidized mitochondrial DNA releasing, and ROS production — is proposed as another upstream signaling event of NLRP3 inflammasome activation (38). We examined and found that echinatin did not affect nigericin-induced mitochondrial damage (Figure 3F). Thus, these findings suggest that the inhibitory effect of echinatin on NLRP3 inflammasome activation is not associated with the K^+^ efflux or mitochondrial damage.

### Echinatin binds to HSP90 and inhibits its ATPase activity, and it disrupts the association of SGT1 and the HSP90-NLRs complex.

**Figure 4:**
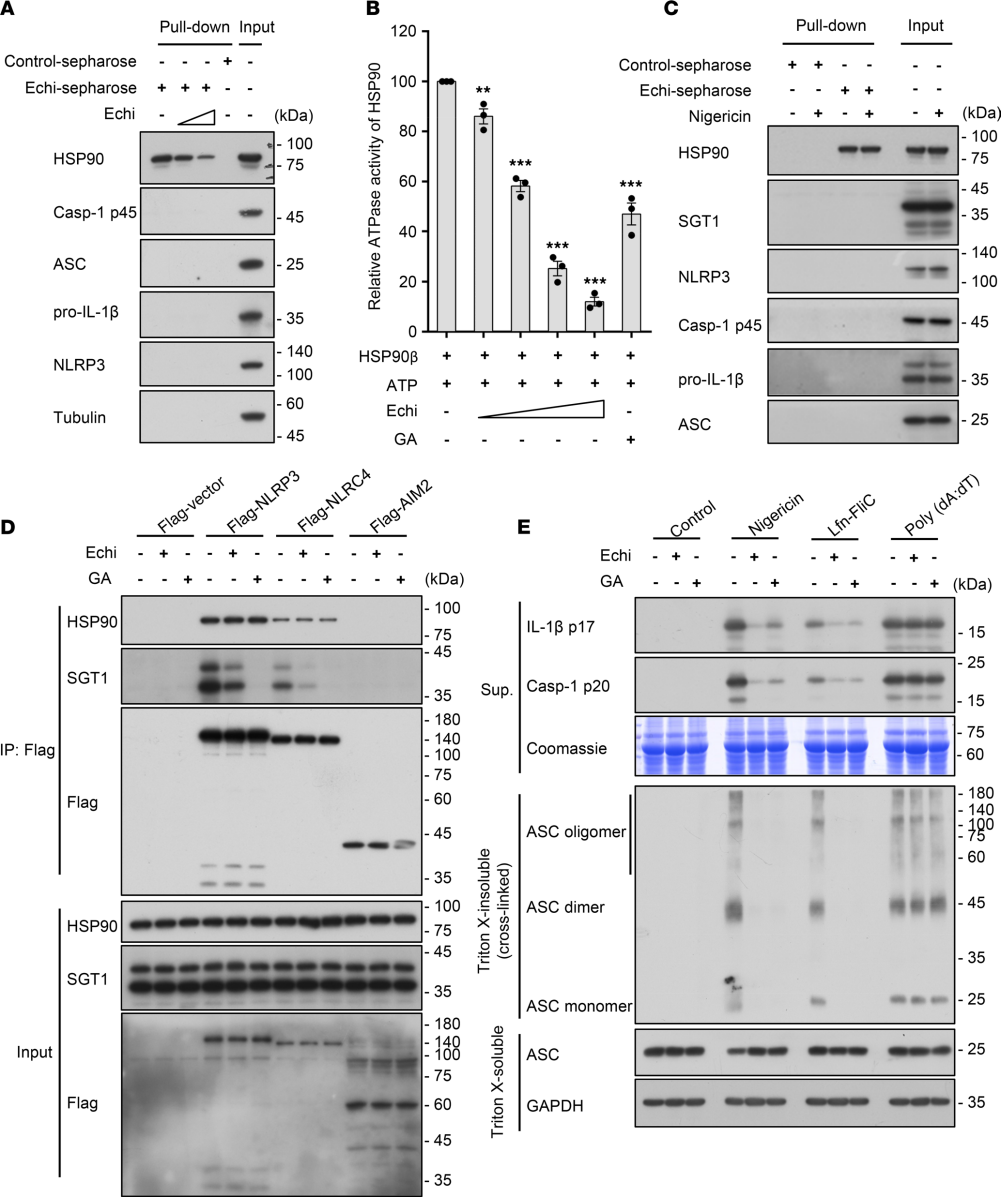
Echinatin binds to HSP90 and inhibits its ATPase activity. (**A**) Cell lysates of LPS-primed BMDMs incubated with echinatin-sepharose and different concentrations of free echinatin (0.4 mM and 0.8 mM). The pull-down samples and input were analyzed by IB. (**B**) Effect of echinatin on the ATPase activity of HSP90β. After incubation HSP90β plus indicated different concentrations of free echinatin (0.25 mM, 0.5 mM, and 1 mM) and geldanamycin (GA, 20 μM), ATP was measured by CellTiter-Glo and normalized to the control. (**C**) Cell lysates of LPS-primed BMDMs with or without nigericin incubated with echinatin-sepharose. The pull-down samples and input were analyzed by IB. (**D**) 293T cells were transfected with indicated plasmids and then treated with vehicle, echinatin (80 μM), and GA (20 μM). Immunoprecipitation was performed with anti–Flag M2 agarose beads; the IB for HSP90, SGT1, and Flag is shown. (**E**) LPS-primed BMDMs were pretreated with vehicle, echinatin (40 μM), or GA (20 μM) and then stimulated with nigericin, Lfn-FliC, or poly(dA:dT). Cleaved caspase-1, production of IL-1β, and cross-linked ASC in the Triton X–insoluble pellet were examined by IB analysis. Data are expressed as mean ± SEM (*n* = 3/group, resulting from 3 independent experiments). One-way ANOVA, followed by Dunnett’s post hoc test, was used to assess the differences of multiple groups (**B**), **P < 0.01, ***P < 0.001 compared with control.

Next, we explored whether echinatin could directly bind to the proteins involved in NLRP3 inflammasome activation. Echinatin conjugated with cyanogen bromide–activated (CNBr-activated) sepharose (echinatin-sepharose) was employed to pull down the echinatin-interacting proteins in the cell lysates of LPS-primed BMDMs, and the relevant proteins were detected by immunoblot analysis. The data show that ASC, NLRP3, and other NLRP3 inflammasome components were not pulled down by echinatin-sepharose (Figure 4A). However, HSP90 — which plays a crucial role in NLRP3 inflammasomes activation (39) — was pulled down (Figure 4A). Moreover, the pull-down of HSP90 by echinatin-sepharose could be competed off by free echinatin (Figure 4A). Previous studies have shown that HSP90 ATPase activity is essential for inflammasome activation (39). Hence, we tested the effect of echinatin on HSP90 ATPase activity and found that echinatin dose-dependently blocked HSP90 ATPase activity (Figure 4B).

In addition, it has been reported that pharmacological inhibition of HSP90 leads to the dissociation of HSP90 client-adaptor SGT1 from HSP90 and NLRs protein, whereas the association of SGT1, HSP90, and NLRs protein is dispensable for the inflammasome activation (39). We first tested whether echinatin affects the interaction between HSP90 and SGT1. The echinatin-interacting proteins were pulled down in the cell lysates of LPS-primed BMDMs treated with nigericin or not, and the relevant proteins were detected by immunoblot analysis. The results showed that the HSP90 client-adaptor SGT1 could not be pulled down with echinatin-sepharose (Figure 4C), suggesting that echinatin broke the interaction between HSP90 and SGT1. Then, we further evaluated the influence of echinatin on the association of SGT1, HSP90, and NLRs protein. HEK-293T cells were transfected with Flag-tagged NLRP3, NLRC4, or AIM2 and then treated with echinatin. Through performing a co-IP assay with anti-Flag M2 beads, we found that HSP90 and SGT1 interacted with tested NLRs members, but not AIM2, and that the HSP90-NLRs interaction is consistent and unaffected. Geldanamycin (GA), the well-characterized HSP90 inhibitor, can block HSP90 ATPase activity and induce the disassociation of SGT1 from HSP90-NLRs (39). Consistent with previous studies, the association of SGT1 with HSP90-NLRs complex was almost completely blocked by GA treatment (Figure 4D). Similar to GA treatment, we found that echinatin also induced the disassociation of SGT1 and the HSP90-NLRs complex (Figure 4D). Consistent with the observation that the SGT1-HSP90-NLRs was disrupted after echinatin or GA treatment, both echinatin and GA treatment were also equally effective in blocking NLRs inflammasome activation, and subsequent caspase-1 activation and pro–IL-1β processing, but not the AIM2 inflammasome (Figure 4E). Taken together, these data suggest that echinatin blocked NLRs inflammasome activation, but not AIM2 inflammasome, by binding to HSP90 and suppressing its ATPase activity and by disrupting the association of SGT1 and the HSP90-NLRs complex.

### Echinatin inhibits NLRP3 inflammasome activation in vivo and ameliorates LPS-induced septic shock.

**Figure 5:**
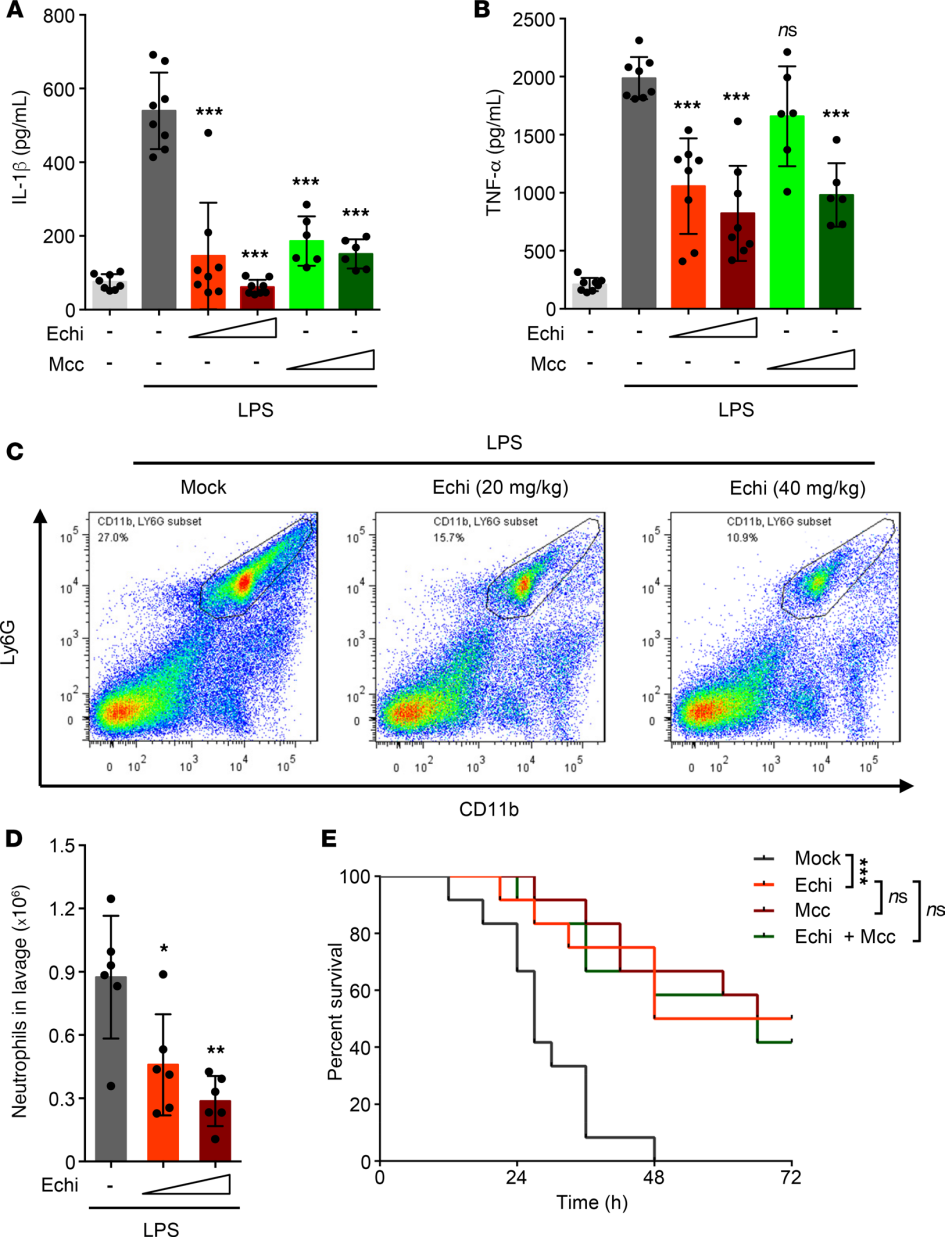
Echinatin inhibits NLRP3 inflammasome activation in vivo and ameliorates LPS-induced septic shock. (**A** and **B**) ELISA of IL-1β (**A**) and TNF-α (**B**) in the serum of mice i.p. injected with LPS (20 mg/kg body weight) in the presence or absence of echinatin (20 mg/kg and 40 mg/kg) and MCC950 (20 mg/kg and 40 mg/kg). (Respectively, mock-PBS [*n* = 8]; mock-LPS [*n* = 8]; 20 mg/kg echinatin-LPS [*n* = 8]; 40 mg/kg echinatin-LPS [*n* = 8]; 20 mg/kg MCC950-LPS [*n* = 6]; 40 mg/kg MCC950-LPS [*n* = 6]). (**C**) Representative FACS plots of neutrophils in the peritoneal cavity from mice i.p. injected with LPS (20 mg/kg body weight) in the presence or absence of echinatin (20 mg/kg and 40 mg/kg). (**D**) FACS analysis of neutrophil numbers in the peritoneal cavity described in **C** (*n* = 6 for each group). (**E**) Survival of WT mice i.p. injected with 20 mg/kg LPS that pretreated with vehicle (*n* = 12), echinatin (40 mg/kg, *n* = 12), MCC950 (40 mg/kg, *n* = 12), or the combination (*n* = 12). Data are expressed as mean ± SD. One-way ANOVA, followed by Dunnett’s post-hoc test, was used to assess the differences of multiple groups. *P < 0.05, **P < 0.01, ***P < 0.001 compared with Mock-LPS (**A**, **B**, and **E**) or to echinatin-LPS (**D**).

We next evaluated the role of echinatin in the activation of the NLRP3 inflammasome in vivo. I.p. injection of LPS could induce NLRP3-dependent IL-1β production and septic shock in mice (40). We pretreated mice with echinatin or MCC950, a best-studied selective inhibitor of NLRP3 (20), and then conducted i.p. injection of LPS. Proinflammatory cytokines and neutrophils in peritoneal lavage fluids were measured. The data show that echinatin effectively attenuated LPS-induced IL-1β and TNF-α production, which was equivalent to the inhibitory effect of MCC950 (Figure 5, A and B). Moreover, consistent with the inhibitory effects of echinatin on proinflammatory cytokines, the proportion and the number of neutrophils in peritoneal lavage cells from mice pretreated with echinatin was also reduced (Figure 5, C and D). Next, we assessed the effect of echinatin on LPS-induced lethality and found that pretreatment with echinatin significantly improved mouse survival compared with the controls (Figure 5E). Interestingly, the mice that combined echinatin and MCC950 administration exhibited the same level of resistance to LPS-mediated lethality compared with echinatin or the well-known NLRP3 inhibitor MCC950 alone (Figure 5E), which suggested that echinatin attenuated LPS-induced lethality efficaciously via targeting NLRP3 inflammasome.

Additionally, we also evaluated the safety of echinatin in vivo. Compared with the control group, mice that received 120 mg/kg dose of echinatin (3 times higher than the dose used for the septic shock experiments) daily for 15 days did not result in any biochemical (such as plasma alanine aminotransferase [ALT] and aspartate aminotransferase [AST] activities, creatinine level), morphological, or weight changes (Supplemental Figure 3, A–E), suggesting that echinatin is well tolerated and safe in mice. Taken together, these results suggest that echinatin is safe and improves the survival of mice suffering from septic shock by inhibiting NLRP3 inﬂammasome.

### Echinatin is efficacious in dextran sodium sulfate–induced (DSS-induced) colitis model.

**Figure 6:**
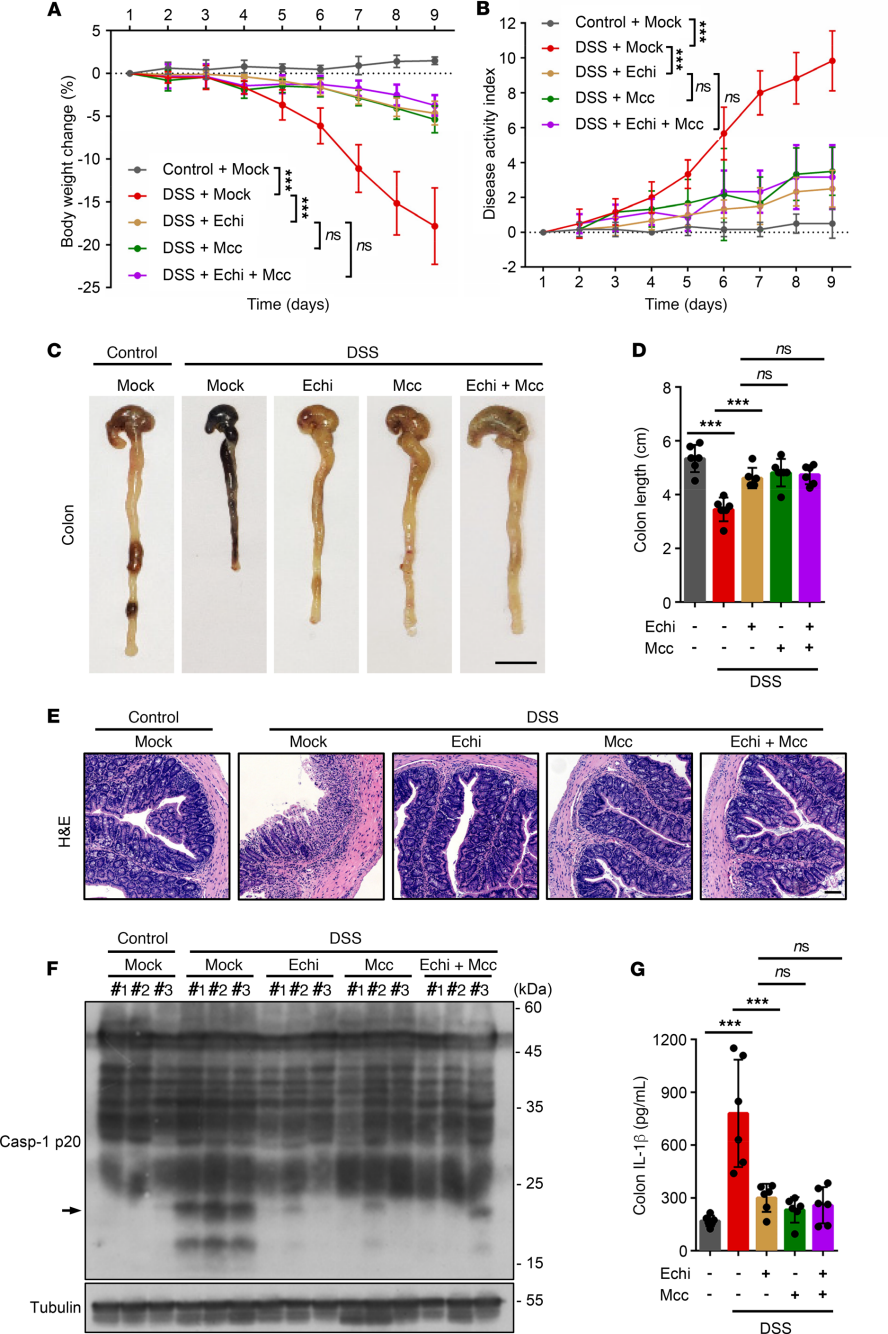
Echinatin is efficacious in DSS-induced colitis model. (**A** and **B**) WT C57BL/6 mice were given 2.5% DSS in the drinking water in the presence or absence of echinatin (40 mg/kg), MCC950 (40 mg/kg), or the combination for 9 days. Body weights (**A**) and disease activity index (**B**) of the mice were measured (*n*=6 for each group). (**C**–**E**) Representative colon images (**C**), the colon lengths (**D**, *n*=6 for each group), and H&E-stained colon sections (**E**) were measured 10 days after treatment with DSS plus vehicle, echinatin (40 mg/kg), MCC950 (40 mg/kg), or the combination. Scale bar: 1 cm (**C**), 200 μm (**E**). (**F** and **G**) Representative IB analysis of active caspase-1 (**F**) and ELISA assay of IL-1β (**G**, *n*=6 for each group) in colon tissues. Data are expressed as mean ± SD. One-way ANOVA, followed by LSD post hoc test, was used to assess the differences of multiple groups (**A**, **B**, **D**, and **G**). ***P < 0.001.

Previous studies have demonstrated that NLRP3 inflammasome is involved in the pathogenesis of colitis (41, 42), and targeting NLRP3 inflammasome has been shown to have definite therapeutic effects in a mouse model of colitis (43–46). To test whether echinatin can block DSS-induced colitis and the activation of NLRP3 in vivo, 8-week-old C57BL/6 male mice were fed with 2.5% DSS in the drinking water for 9 days and administered with echinatin, MCC950, or the combination by i.p. daily. The results showed that echinatin, MCC950, or echinatin plus MCC950 treatment significantly protected against body weight loss and decreased the disease activity index (DAI) scores (Figure 6, A and B), which are crucial parameters indicating the severity of colitis (47). At the same time, the marked improvement in the severity of colitis in these compound-administered mice was confirmed by the restoration of DSS-induced shortened colon length (Figure 6, C and D). Interestingly, compared with the administration of echinatin or MCC950 alone, there is no significant difference of the above pathological indicators after echinatin plus MCC950 treatment. Moreover, histopathological analysis of the mouse colons showed that DSS elicited colonic inflammation, including disruption of mucosal barrier and inflammatory cell infiltration, which were also reversed by echinatin, MCC950, or echinatin plus MCC950 administration (Figure 6E). Next, we investigated whether administration of these compounds affected NLRP3 inflammasome activation in the experimental colitis model. As expected, we found that all 3 types of interventions significantly attenuated caspase-1 activation and IL-1β production in the colon tissue of DSS-treated mice (Figure 6, F and G). Similarly, no significant improvements were observed in the combination (Figure 6, F and G). Taken together, these results indicate that echinatin attenuates NLRP3 inflammasome activation and reverses the pathological process of DSS-induced colitis.

### Echinatin exhibits a therapeutic effect in NASH model.

**Figure 7:**
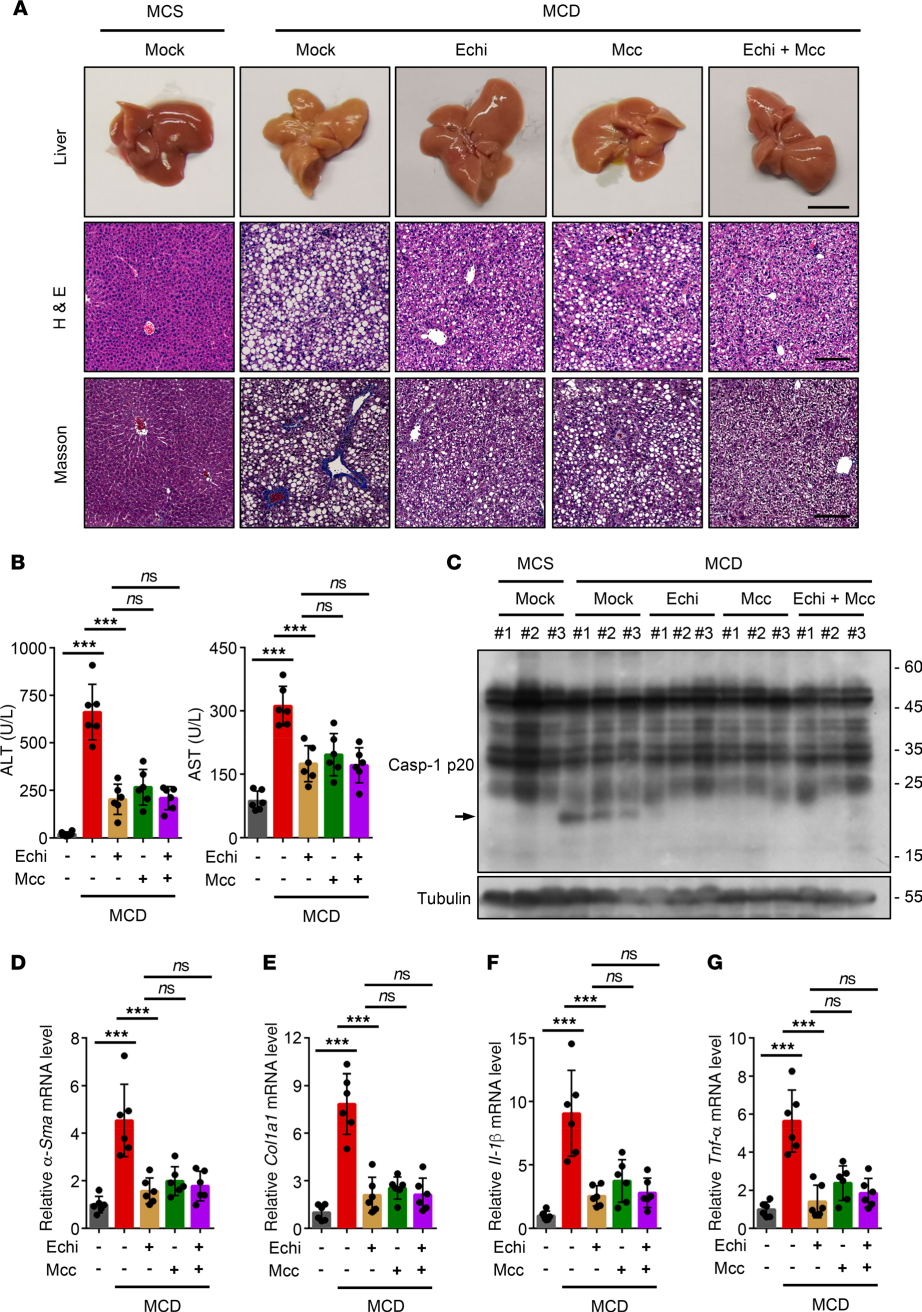
Echinatin exhibits therapeutic effect in nonalcoholic steatohepatitis (NASH) model. (**A**) Representative liver images, H&E-stained, and Masson-stained liver sections are shown from the mice fed MCD or MCS diets in the presence or absence of echinatin (Echi; 40 mg/kg), MCC950 (40 mg/kg), or Echi plus MCC950. Scale bar: 1 cm (top row), 200 μm (bottom 2 rows). (**B**) The activity of plasma ALT and AST were measured as described in **A** (*n*=6 for each group). (**C**) Representative IB analysis of active caspase-1 level in liver tissues described in **A**. (**D**–**G**) Hepatic *α-Sma* (**D**)*, Col1a1* (**E**)*, Il-1β* (**F**), and *Tnf-α* (**G**) mRNA were measured from the mice described in **A** (*n*=6 for each group). Data are expressed as mean ± SD. One-way ANOVA, followed by LSD post hoc test, was used to assess the differences of multiple groups (**B** and **D**–**G**). ***P < 0.001.

It has also been reported that NLRP3 inflammasome is regarded as an important contributor to methionine- and choline-deficient (MCD) diet–induced nonalcoholic steatohepatitis (NASH) (48), and blockade of NLRP3 inflammasome activation can reduce liver inflammation and fibrosis, as well as improve NASH pathology (49). We next tested the protective effects of echinatin on the MCD dietary mouse model, which is a mouse NASH model that shows several features of human NASH (50, 51), such as hepatic steatosis, inflammatory cell infiltration, and fibrosis (50). Mice were fed with a MCD diet for 6 weeks and then received a 40 mg/kg dose of echinatin and/or MCC950 daily for the last 5 weeks. MCC950 treatment alone was employed as a positive control (49). Compared with the mice fed with a methionine-choline–supplemented (MCS) diet, the MCD diet–fed mice exhibited obvious liver morphological changes, hepatic steatosis, ballooning, fibrosis (Figure 7A), and remarkably higher levels of plasma ALT and AST (Figure 7B). As expected, the aforementioned disease phenotypes were robustly alleviated by echinatin or MCC950 treatment. Also, we found that the therapeutic effect of the combined administration on MCD diet–induced NASH pathology and fibrosis is similar to that of echinatin or the well-characterized NLRP3 inhibitor MCC950 alone (Figure 7, A and B).

To test whether echinatin blocks NLRP3 inflammasome activation in the livers with NASH, the expression of active caspase-1 in liver tissue treated with or without echinatin was analyzed. As shown in Figure 7C, the results indicated that caspase-1 activation in the liver of mice fed with a MCD diet was suppressed by echinatin or MCC950 treatment. In addition, the transcription level of genes involved in hepatic fibrogenesis was evaluated. Both echinatin and MCC950 treatment markedly reduced the mRNA levels of α–smooth muscle actin (α-SMA) and α–1 type I collagen (Col1a1) in the livers of MCD diet–fed mice (Figure 7, D and E). Similar results were observed in mRNA expression of proinflammatory genes IL-1β and TNF-α (Figure 7, F and G). We also evaluated whether echinatin plus MCC950 treatment had better beneficial effect on the hepatic expression of active caspase-1 and the aforementioned profibrotic markers, and we found that the combination showed similar beneficial effects in the liver compared with echinatin or MCC950 treatment alone (Figure 7, D–G). Overall, these results suggest that echinatin alleviates liver inflammation and improves the NASH pathology in an experimental NASH mouse model via inhibition of NLRP3 inflammasome.

## Discussion

NLRP3 inflammasome has been reported to be involved in a variety of inflammatory diseases including T2D, gout, and Alzheimer’s disease, as well as colitis and NASH (5, 43, 51). Given its role in pathogenesis, blockade of NLRP3 inflammasome is regarded as a promising intervening strategy for the treatment of related human complex diseases (5, 43, 51). Therefore, the study on NLRP3 inflammasome has attracted considerable attention, and significant progresses have been made in recent years. Several small molecules that pharmacologically target NLRP3 have been reported, including tranilast (52), MCC950 (20), OLT1177 (53), CY-09 (54), oridonin (55), cardamonin (56, 57), and isoliquiritigenin (19); these exhibited potentially therapeutic effects in animal models of NLRP3-driven diseases. Recently, it has been reported that tranilast, which is an old and safe clinic drug, could selectively and potently inhibit the NLRP3 inflammasome activation and is effective in mouse models of gout and T2D (52), while the therapeutic effect of tranilast needs to be further evaluated in clinical trials. MCC950 has been considered as the best-studied selective inhibitor of NLRP3 and is efficacious in a variety of immunopathological mouse models of NLRP3-driven diseases, such as CAPS, colitis, and steatohepatitis, but it was suspended in a phase II clinical trial for treating rheumatoid arthritis because of the hepatotoxicity (5). Currently, OLT1177 is the only drug tested in a phase II clinical trial (14). Therefore, it is urgent to develop available NLRP3 inhibitors approved for therapeutic use.

Echinatin, the bioactive constituent from licorice, has shown antioxidant and antiinflammatory activities (23, 25), as well as potential therapeutic properties for psoriasis in the European patent EP0998939A1. Our results demonstrate that echinatin possesses remarkable therapeutic efficacy in several experimental mouse models, such as LPS-mediated septic shock, DSS-induced acute colitis, and MCD diet–induced NASH. As shown in Figure 5, Figure 6, and Figure 7, compared with echinatin or MCC950 treatment alone, the combined treatment exhibited similar protective effects in lethal LPS-induced sepsis, DSS-induced colitis, and MCD diet–induced NASH. Taken together, these results suggest that echinatin administration ameliorated aforementioned inflammatory diseases by suppressing NLRP3 inflammasome activation. Of note, echinatin has been shown to be safe because mice receiving 3 times higher than the dose used for all examined mouse models daily for 15 days do not exhibit any biochemical, morphological, or weight changes. Further studies are required to evaluate the therapeutic effect on human NLRP3–driven diseases in clinical trials. Additionally, licorice, the original source of echinatin, is one of the most commonly used traditional herbal medicines with various biological properties, including hepatoprotection, antiinflammation, and antiallergy properties (19). However, the precise mechanisms and molecular target are yet largely unexplored. Our findings, therefore, contribute to a better understanding of the pharmacological effect of licorice from the perspective of its bioactive ingredients.

HSP90 is a multifunctional molecular chaperone that regulates the stability and the activation of other proteins (clients) related to signal transduction, protein trafficking, immunity, and receptor maturation (45). It has been reported that pharmacological inhibition of HSP90 suppresses NLRs inflammasome activity (37). Through identifying the target protein of echinatin, we found that echinatin binds to HSP90 and inhibits its ATPase activity. Exposure to echinatin induces the dissociation of SGT1 from HSP90, which is crucial for inflammasome activation, but the association of the HSP90-NLRs complex was not altered under the same conditions. Our data indicate that the inhibitory effect of echinatin on NLRP3 inflammasome may be due to the dissociation of SGT1 from HSP90, the suppression of HSP90 ATPase activity, and the interruption of the association between SGT1 and HSP90-NLRP3. The above factors led to the suppression of the assembly and subsequent activation of NLRP3 inflammasome.

In addition to NLRP3, a number of other innate immune sensors could also assemble inflammasomes, such as flagellin sensor NLRC4 and double-stranded DNA sensor AIM2. The underlying mechanisms that mediate or regulate inflammasome activation remain unclear. Through evaluating the effect of echinatin on activity of different types of inflammasome, we found that echinatin only blocked NLRs inflammasome activation, including the NLRP3 and NLRC4 inflammasome, but not double-stranded DNA–triggered AIM2 inflammasome activation. Further study found that only NLRs sensor (NLRP3 and NLRC4) interact with HSP90-SGT1, but not AIM2. These finding suggest that HSP90-SGT1 is the common regulator of NLRs inflammasome, and the different regulatory roles of echinatin on NLRs and AIM2 inflammasome might be due to the domain structure differences between NLRs and AIM2 — probably the LRR domain. In support of this hypothesis, it has been reported that the characteristic domain of NLRs sensors are the nucleotide-binding and oligomerization domain (NACHT) and leucine-rich repeat (LRR) domains, whereas AIM2 does not possess the above-mentioned domain and is composed of a pyrin domain (PYD) and a DNA-binding HIN200 domain. Meanwhile, previous studies have shown the NLRs LRR domain is essential for the binding of NLRs to the HSP90-SGT1 complex (39, 58). In addition, these findings suggest that HSP90-SGT1 is the common regulator of NLRs inflammasome but not the AIM2 inflammasome.

In this study, we uncovered a potentially novel and potent antagonist of NLRP3 inflammasome, namely echinatin. Echinatin exerts markedly inhibitory effects on NLRP3 inflammasome activation in vitro and in vivo. Our results demonstrate that echinatin directly binds to HSP90 and inhibits its ATPase activity, disrupts the HSP90-SGT1 association, and prevents the interaction between SGT1 and the HSP90-NLRs complex; subsequently, it suppresses NLRs inflammasome activation. Importantly, echinatin displays significantly therapeutic effects in several mouse models of NLRP3-mediated diseases and may be used as a favorable candidate approach for therapeutic interventions in NLRP3 inflammasome–driven diseases.

## Methods

### Study design.

The aim of this study was to evaluate the therapeutic effect of echinatin on NLRP3 inflammasome–driven diseases and clarify the related mechanisms. We examined the effect of echinatin on NLRP3 inflammasome activation and investigated the related mechanisms in mouse BMDMs and hPBMCs using immunoblot, ELISA, immunoprecipitation, pull-down assay, and histology staining. This was combined with several experimental mouse models of NLRP3 inflammasome–driven diseases to evaluate its therapeutic effects. For in vitro studies, experiments consisted of at least 3 independent experiments with biological duplicates. For in vivo studies, animals were randomly assigned to treatment groups and humanely euthanized at defined study end points.

### Animals.

C57BL/6 mice were purchased from SPF Biotechnology Co. Ltd. Mice were housed under pathogen-free conditions. All of the studies used age- and sex-matched mice.

### Reagents and antibodies.

Adenosine triphosphate (ATP, A2383) and dimethyl sulfoxide (DMSO, D2650) were purchased from MilliporeSigma. MSU (tlrl-msu), SiO2 (tlrl-sio), poly (dA:dT) (tlrl-patn), Pam3CSK4 (tlrl-pms), PMA (tlrl-pma), and ultrapure LPS were purchased from Invivogen. Nigericin (ab120494) and DSS (ab141274) were from Abcam. Echinatin (T3928) was from TargetMol. Lfn-FliC was provided by Tao Li from National Center of Biomedical Analysis (Beijing, China). Caspase-Glo 1 Inflammasome Assay (G9951), CytoTox 96 Non-Radioactive Cytotoxicity Assay (G1780), and CellTiter-Glo Luminescent Cell Viability Assay (G7572) were from Promega. MitoTracker and MitoSOX were from Invitrogen. Anti–mouse caspase-1 (1:1000, AG-20B-0042) and anti-NLRP3 (1:2000, AG-20B-0014) were bought from Adipogen. Anti–mouse cleaved caspase-11 (1:1000, ab180673) was purchased from Abcam. Anti–human cleaved IL-1β (1:2000, 12242), anti–human caspase-1 (1:2000, 4199S), anti–mouse IL-1β (1:2000, AF-401-NA), and anti-NLRP3 (1:2000, 15101S) were obtained from Cell Signaling Technology. Anti–mouse IL-1β (1:2000, AF-401-NA) was from R&D Systems. Anti-ASC (1:1000, sc-22514-R) was from Santa Cruz Biotechnology Inc. Anti-GAPDH (1:5000, 60004-1-1g), anti-tubulin (1:5000, 66031-1-1g), anti-Flag (1:2000, 20543-1-AP), anti-HSP90 (1:3000, 13171-1-AP), and anti-SGT1 (1:2000, 11675-1-AP) were purchased from Proteintech. Salmonella is a gift from Tao Li.

### Plasmids.

pCMV-Flag-Vector, pCMV-NLRP3-Flag, and pcDNA3.0-Flag-AIM2 were provided by Tao Li from National Center of Biomedical Analysis (Beijing, China). pcDNA3.0-Flag-NLRC4 was from Bioworld Technology.

### Cell culture.

PBMCs were isolated from peripheral blood from informed healthy volunteers and were cultured in RPMI-1640 medium (CM10040, Macgene). Human THP-1 cells and HEK-293T cells were provided by Tao Li (National Center of Biomedical Analysis) and have been tested to be mycoplasma free by PCR (MP0025, Merck). THP-1 cells were grown in RPMI-1640 medium and differentiated with 100 nM PMA overnight. HEK-293T was cultured in DMEM medium. All cell lines were grown in a 5% CO_2_ humidified incubator at 37°C (HEARcell 150i, Thermo Fisher Scientific).

### Immunoprecipitation and pull-down assay.

The cell lysates from HEK-293T cells, which have been transfected with Flag-tagged plasmids (Flag-Vector, Flag-NLRP3, Flag-NLRC4, Flag-AIM2) for 24 hours and then treated with compounds for 6 hours, were collected and lysed with lysis buffer (50 mM Tris, pH 7.8, 5 0 mM NaCl, 0.1% [vol/vol] Nonidet-P40, 5 mM EDTA, and 10% [vol/vol] glycerol) containing complete protease inhibitor cocktail (TargetMol, C0001), and then centrifuged at and then centrifuged at 13,200*g* for 15 minutes at 4°C. The supernatants were immunoprecipitated with anti-Flag M2 affinity beads (A2220, MilliporeSigma) according to the manufacturer’s instruction. Cell lysates or immunoprecipitates were separated by SDS-PAGE and analyzed with immunoblotting.

### Echinatin pull-down assays.

Echinatin was conjugated with CNBr-activated Sepharose 4B (GE Healthcare). BMDMs were lysed with lysis buffer containing complete protease inhibitor cocktail (TargetMol, C0001), followed by centrifugation at 12,000*g* for 20 minutes at 4°C. The supernatants were incubated with echinatin-conjugated Sepharose 4B at 4°C overnight. The beads were then washed 6 times with lysis buffer. The proteins pulled down were separated by SDS-PAGE and analyzed by immunoblotting.

### Inflammasome activation.

To induce inflammasomes activation, BMDMs at 1 × 10^6^ cells/mL were seeded in 24-well plates overnight. Then, the medium was replaced the following day, and the cells were stimulated with 50 ng/mL LPS or 400ng/mL Pam3CSK4 for 4 hours. The cells were treated with echinatin for 1 hour and then stimulated as follows: 7.5 μM nigericin for 30 minutes, 5 mM ATP for 45 minutes, 200 mg/mL monosodium urate (MSU) for 6 hours, or 2 mg/mL PA in combination with 200 ng/mL Lfn-FliC for 4 hours. A total of 1 μg/mL ultra-LPS, 2 μg/mL poly(I:C), or 2 μg/mL poly(dA:dT) was transfected into BMDMs with Lipofectamine 2000 for 4 hours. PBMCs cells were seeded in 6-well culture dishes (6 × 10^6^ cells/mL) overnight. Cells were then stimulated with 10 μM nigericin for 1 hour.

### Caspase-1 activity assay.

The protocol for the caspase-1 activity assay has been described previously (59, 60). Briefly, the Caspase-Glo 1 reagent was prepared according to the instructions. The 1:1 ratio of Caspase-Glo 1 Reagent volume/sample volume was used to measure released caspase-1 activity in cell culture medium.

### ASC oligomerization assay.

The assay for ASC oligomerization has been described previously. Cells were lysed with Triton Buffer (50 mM Tris-HCl [pH 7.5], 150 mM NaCl, 0.5% Triton X-100, and EDTA-free protease inhibitor cocktail) and then centrifuged at 6000*g* at 4°C for 15 minutes. The supernatant and pellet fractions were referred to as the Triton X–soluble and Triton X–insoluble fractions, respectively. For ASC oligomer cross-linking, the Triton X-100–insoluble fractions were washed and resuspended in 200 μL PBS and cross-linked for 30 minutes at 37°C with 2 mM DSS. The pellets were centrifuged for 15 minutes at 6000*g* at 4°C and then collected and dissolved in 1× SDS loading buffer for immunoblot analysis.

### Intracellular K^+^ measurement.

The protocol for the measurement of intracellular potassium has been described previously (61). Briefly, BMDMs were seeded at 1 × 10^6^ cells/mL with 1.0 mL medium overnight in 12-well plates. After being primed with 50 ng/mL LPS for 4 hours, cells were treated with echinatin and then stimulated with nigericin. Culture medium was thoroughly aspirated and washed 3 times in saline, and ultrapure HNO_3_ was added to lyse the cells. Then, samples were collected and boiled for 30 minutes at 100°C. Intracellular K^+^ measurements were performed by inductively coupled plasma optical emission spectrometry.

### HSP90β ATPase activity assay.

HSP90β ATPase activity was performed in the reactions (33 mM HEPES [pH7.2], 30 mM NaCl, 5 mM MgCl_2_, 1 mM DTT, 1 μM ATP) with or without indicated compounds at 37°C for 1 hour; then, the CellTiter-Glo Assay was employed to measure the quantitation of ATP according to the manufacturer’s instructions, and luminescence was recorded.

### LPS-induced systemic inflammation.

Eight-week-old mice were injected i.p. with echinatin (20 or 40 mg/kg) for 1 hour and were then injected i.p. with LPS (20 mg/kg) (0111:B4; MilliporeSigma). After 2 hours, the mice were sacrificed, and the serum samples were collected; 10 mL of ice-cold PBS was used to wash the peritoneal cavities. The levels of plasma IL-1β and TNF-α were measured by ELISA. The polymorphonuclear neutrophils in peritoneal lavage fluid was analyzed by flow cytometry by staining Ly6G and CD11b. For endotoxic shock model, 8-week-old mice were injected with echinatin (20 or 40 mg/kg) i.p. for 1 hour and then injected with LPS (20 mg/kg) i.p. Mice were monitored for lethality for 3 days.

### Toxicology.

Eight-week-old male and female C57BL/6 mice were injected with echinatin (120 mg/kg) or control solution i.p. daily for 15 days. Body weight was measured every day, and different organs were collected on the 16th day. Serum alanine aminotransferase (53), aspartate transaminase (AST), and creatinine were determined using the commercially available assay kit (Nanjing Jiancheng Bioengineering Institute) according to the manufacturer’s instructions.

### DSS-induced colitis.

Colitis was induced in 8-week-old C57BL/6 male mice with 2.5% DSS (MP Biomedicals) dissolved in drinking water for 9 days. Mice were injected i.p. with echinatin (40 mg/kg) in 5% DMSO in corn oil daily. Control animals received 5% DMSO in corn oil i.p. daily. The DAI is calculated by adding individual scores of diarrhea, bloody stool, and the loss in body weight, and it was used to evaluate and quantify the severity of intestinal damage after DSS administration in our current experiment. The detailed scoring system of DAI is as follows: body weight loss percent relative to day 1 (0, no weight loss; 1, 1%–5 %; 2, 6%–10 %; 3, 11%–20%; 4, >20%); diarrhea (0, normal; 1–2, loose stools; 3–4, watery diarrhea); bloody stool (0, normal stool; 1–2, slight bleeding; 3–4, gross bleeding). The process was performed by a single-blind physician with extensive experience in experimental gastroenterology research.

### MCD diet–induced steatohepatitis and fibrosis.

Groups (*n*=6) of 8-week-old male C57BL/6 mice were fed with MCD diet (TP-3001, TROPHIC Animal Feed High-Tech Co. Ltd.) for 6 weeks; controls were fed with the identical MCS diet (TP-3001c, TROPHIC Animal Feed High-Tech Co. Ltd.) according to the manufacturer’s instructions. During the last 5 weeks, MCD diet–fed mice and MCS controls were divided into groups treated with vehicle, echinatin (40 mg/kg), MCC950 (40 mg/kg), or the combination in 5% DMSO in corn oil daily up to 6 weeks by i.p. At the end of experiments, mice were anesthetized, and liver and serum were collected.

### Statistics.

Statistical calculations were carried out using GraphPad Prism 6.0 (GraphPad Software). All of the experimental data were expressed as the mean ± SEM or the mean ± SD. Statistical comparisons were carried out using an unpaired, 2-tailed *t* test between only 2 groups. One-way ANOVA, followed by Dunnett’s or least significant difference (LSD) post hoc test, was used to assess the differences of multi-groups. Differences with *P* < 0.05 were statistically significant. The difference was considered statistically significant at *P < 0.05, **P < 0.01, and ***P < 0.001.

### Study approval.

All animal experiment procedures were approved by the animal care and use committees of the Fifth Medical Center of Chinese PLA General Hospital. The adult peripheral blood samples were obtained from 3 healthy donors at the Fifth Medical Center of Chinese PLA General Hospital, and the experimental protocols were performed according to the approved guidelines established by the Institutional Human Research Subjects Protection Committee of the Ethics Committee of the Fifth Medical Center of Chinese PLA General Hospital.

## Author contributions

GX, SBF, ZFB, and XHX conceived the project, designed and supervised the experiments, and interpreted results. GX, XYZ, ZFB, and ZLW wrote and revised the manuscript with all other authors. PZ, JBW, MN, and YMG assisted in the study design and helped analyzed the data. GX, SBF, and ZLW performed most of the experiments and data analysis, together with XYZ, PZ, WS, NQ, YYC, and CYW. GX and ZLW collected the human plasma samples. ZLW, WS, NQ, YYC, and CYW helped perform the LPS-induced septic shock animal experiment, the MCD diet–induced NASH, and the DSS-induced colitis mouse model.

## Supplementary Material

Supplemental data

## Version 1 12/22/2020

In-Press Preview

## Version 2 01/25/2021

Electronic publication
