# Supplementary material for: Echinatin effectively protects against NLRP3 inflammasome–driven diseases by targeting HSP90
Source: JCI Insight. 2021 Jan 25;6(2):e134601. doi: 10.1172/jci.insight.134601 (PMC7934863; doi:10.1172/jci.insight.134601)
Supplement: Supplemental data [file jciinsight-6-134601-s204.pdf]

Figures

Figure 1. Echinatin inhibits NLRP3 inflammasome activation in mouse BMDMs and human PBMCs

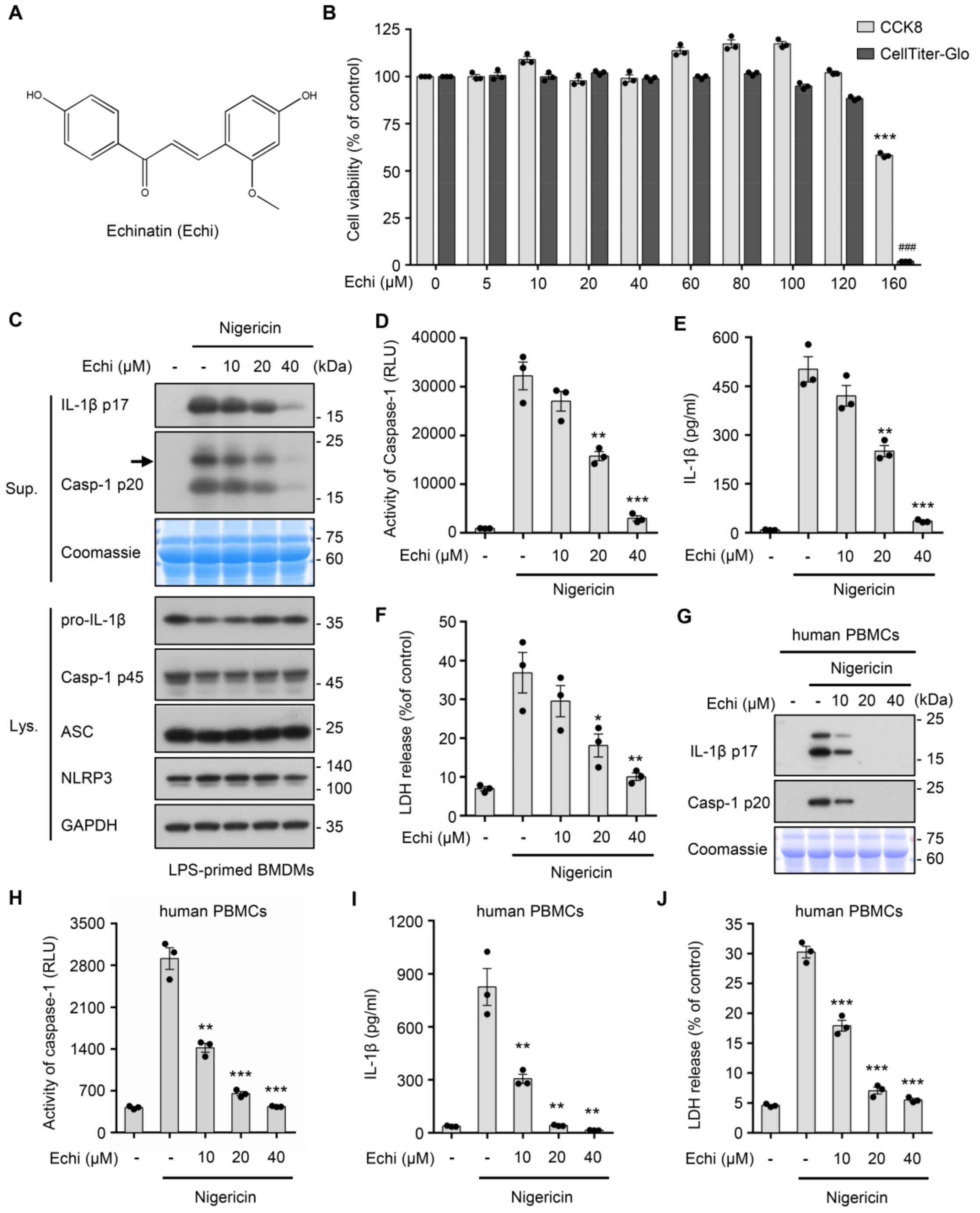

(A) The structure of echinatin (Echi) is shown.

(B) Cell viability of BMDMs treated with indicated dose of echinatin were assessed using Cell Counting Kit-8 (CCK-8) or CellTiter-Glo® Assay which is based on quantitation of ATP.

(C-F) LPS-primed BMDMs were pretreated with various doses of echinatin and then stimulated with nigericin, cleaved caspase-1 and production of IL-1β were examined by immunoblotting (IB) analysis (C), activity of caspase-1 (D), secretion of IL-1β (E) and LDH (F) in SN were assessed.

(G-J) LPS-primed human PBMCs were pretreated with various doses of echinatin and then stimulated with nigericin, cleaved caspase-1 and production of IL-1β were examined by IB analysis (G), activity of caspase-1 (H), secretion of IL-1β (I) and LDH (J) in SN were assessed.

Data are expressed as mean ± SEM (n = 3/group, resulting from three independent experiments). One-way ANOVA followed by Dunnett's post-hoc test was used to assess the differences of multi-groups (B, D-F, H-J). $^{*}p < 0.05$, $^{**}p < 0.01$, $^{***}p < 0.001$, $^{\#\#\#}p < 0.001$ compared to control (B) or control with stimulated (D-F, H-J).

Figure 2. Echinatin suppresses multiple agonists-mediated NLRP3 inflammasome activation and assembly

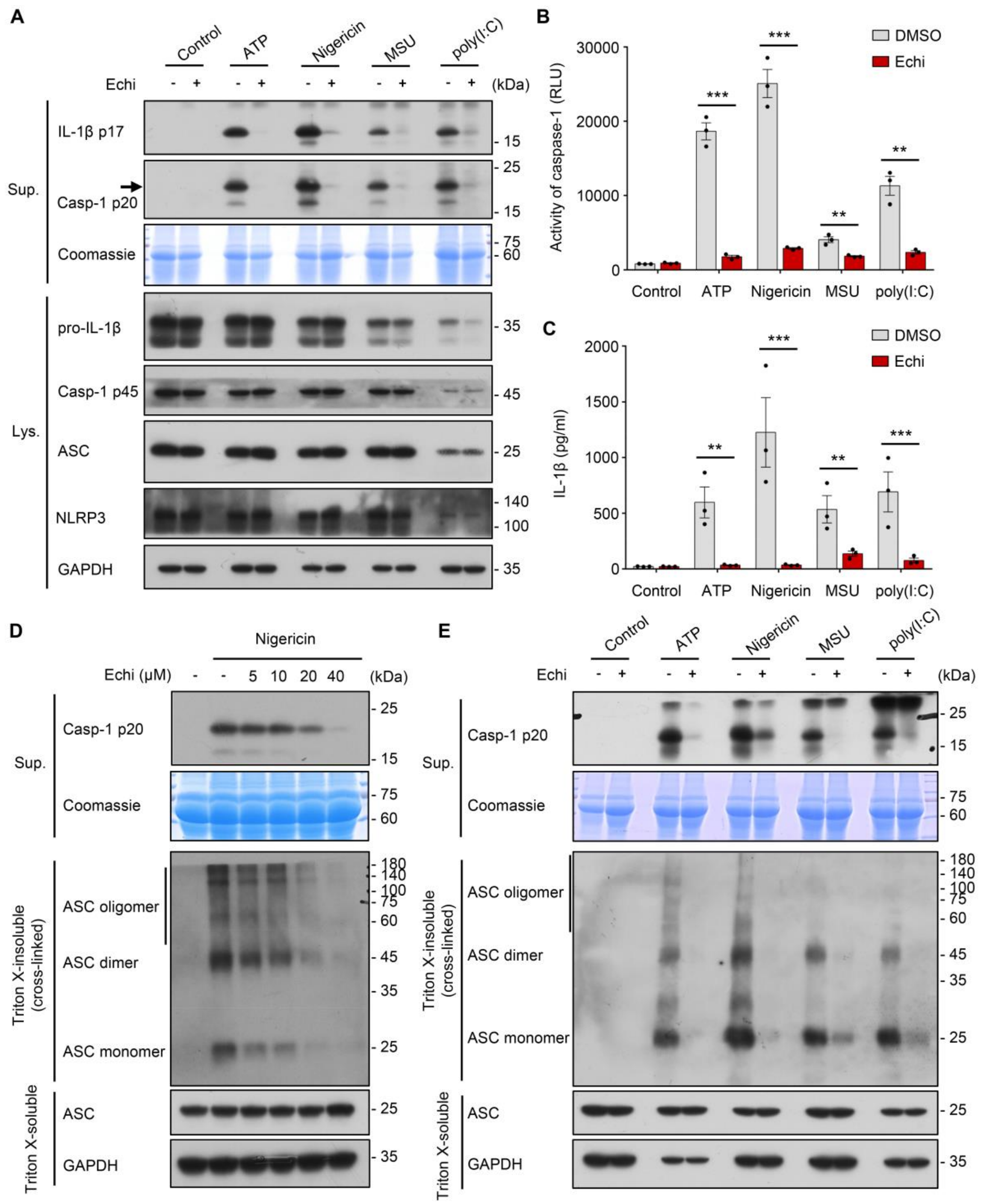

(A-C) LPS-primed BMDMs were pretreated with echinatin (40 μM) or vehicle and then stimulated with ATP, nigericin, MSU and poly(I:C), cleaved caspase-1 and production of IL-1β were examined by IB analysis (A), activity of caspase-1 (B) and secretion of IL-1β (C) in SN were assessed.

(D) LPS-primed BMDMs were pretreated with indicated dose of echinatin and stimulated with nigericin, IB analysis was used to detect cross-linked ASC in the Triton X-insoluble pellet.

(E) IB analysis of cross-linked ASC in the Triton X-insoluble pellet from LPS-primed BMDMs pretreated with echinatin (40 μM) or vehicle and then stimulated with ATP, nigericin, MSU and poly(I:C).

Data are expressed as mean ± SEM (n = 3/group, resulting from three independent experiments). Statistics differences were analyzed by unpaired t test (B, C). $^{**}p < 0.01$, $^{***}p < 0.001$.

Figure 3. Echinatin does not directly target the ASC oligomerization, as well as does not block K^+ efflux or mitochondrial damage

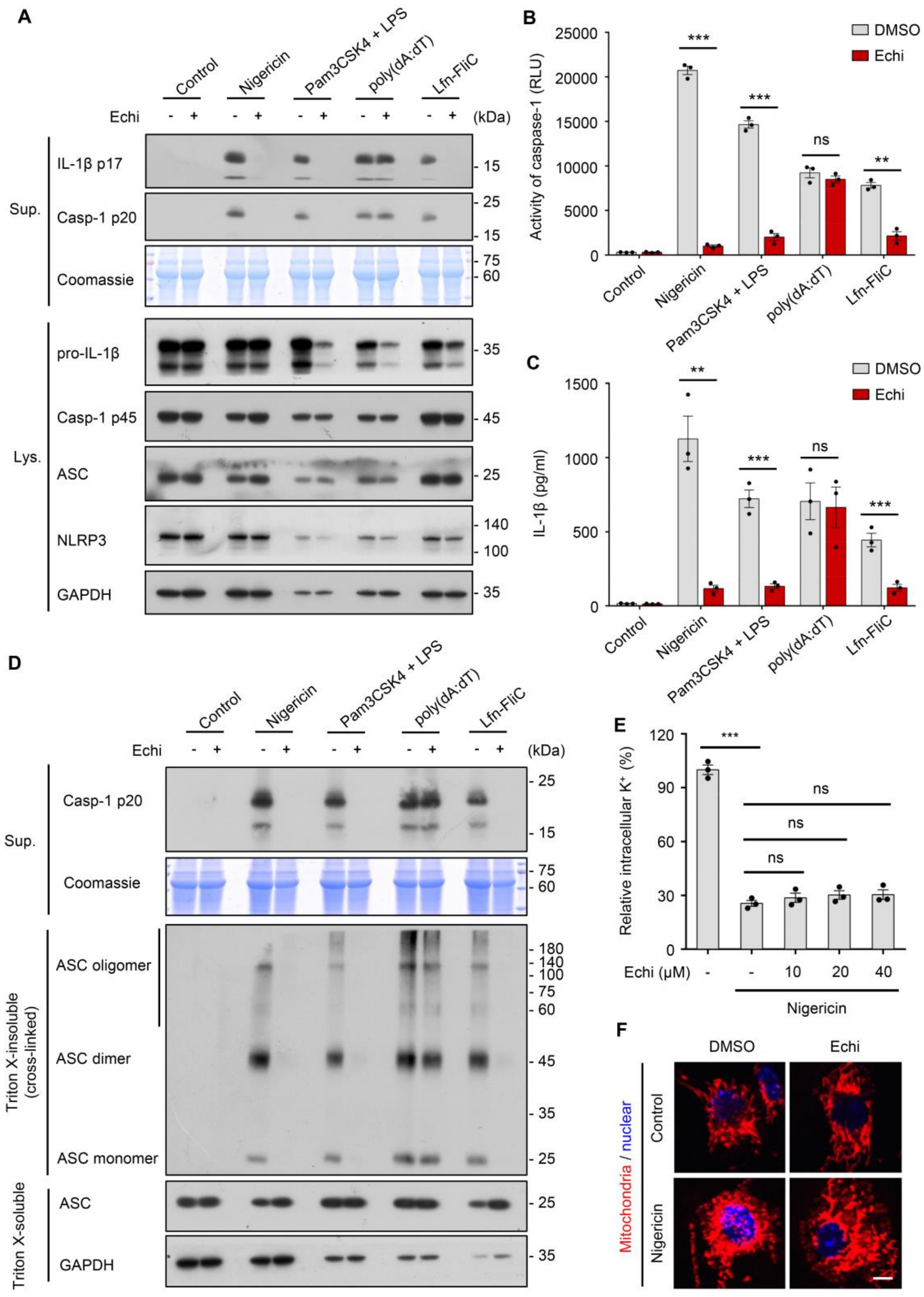

(A-C) LPS-primed BMDMs were pretreated with echinatin (40 μM) or vehicle and then stimulated with nigericin, poly(dA:dT) and Lfn-FliC, or Pam3CSK4-primed BMDMs were pretreated with echinatin (40 μM) or vehicle and then stimulated with transfected LPS. Cleaved caspase-1 and production of IL-1β were examined by IB analysis (A), activity of caspase-1 (B) and secretion of IL-1β (C) in SN were assessed.

(D) IB analysis of cross-linked ASC in the Triton X-insoluble pellet from LPS-primed BMDMs pretreated with echinatin (40 μM) or vehicle and then stimulated with nigericin, poly(dA:dT) and Lfn-FliC, or Pam3CSK4-primed BMDMs were pretreated with echinatin (40 μM) or vehicle and then stimulated with transfected LPS.

(E) Qualification of intracellular potassium in LPS-primed BMDMs pretreated with indicated dose of echinatin and stimulated with nigericin.

(F) Staining with MitoTracker red in LPS-primed BMDMs pretreated with echinatin (40 μM) or vehicle and then stimulated with nigericin. Scale bar: 5 μm.

Data are expressed as mean ± SEM (n = 3/group, resulting from three independent experiments). Statistics differences were analyzed by unpaired t test (B, C) or one-way ANOVA followed by Dunnett's post-hoc test (E). **$p < 0.01$, ***$p < 0.001$, ns: not significant.

Figure 4. Echinatin binds to HSP90 and inhibits its ATPase activity

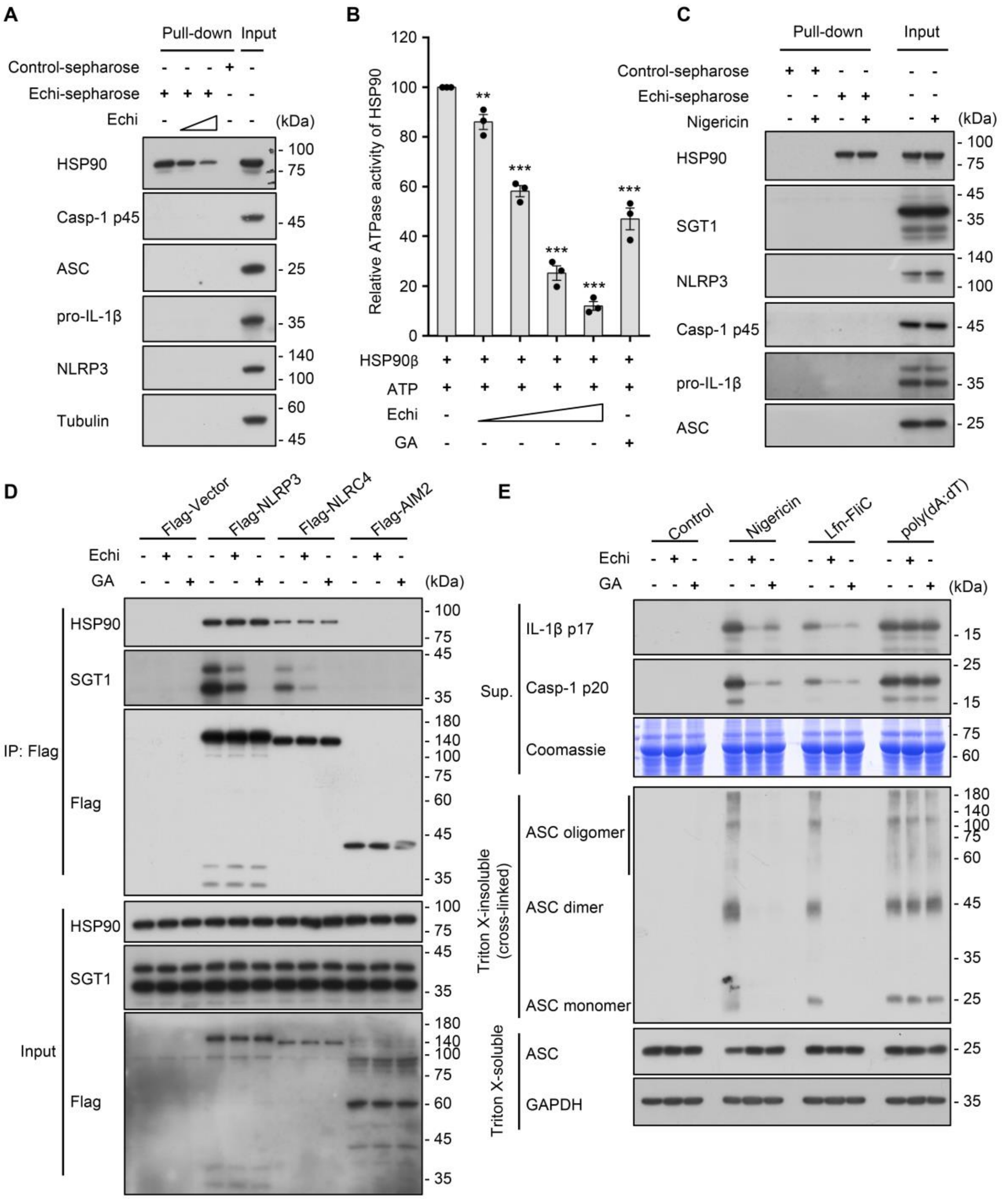

(A) Cell lysates of LPS-primed BMDMs incubated with echinatin-sepharose and different concentrations of free echinatin (0.4 mM and 0.8 mM). The pull-down samples and input were analyzed by IB.

(B) Effect of echinatin on the ATPase activity of HSP90β. After incubation HSP90β plus indicated different concentrations of free echinatin (0.25 mM, 0.5 mM and 1 mM) and geldanamycin (GA, 20 μM), ATP was measured by Cell Titer Glo and normalized to the control.

(C) Cell lysates of LPS-primed BMDMs with or without nigericin incubated with echinatin-sepharose. The pull-down samples and input were analyzed by IB.

(D) 293T cells were transfected with indicated plasmids and then treated with vehicle, echinatin (80 μM) and GA (20 μM), respectively. Immunoprecipitation was performed with anti-flag M2 agarose beads, the IB for HSP90, SGT1 and Flag was shown.

(E) LPS-primed BMDMs were pretreated with vehicle, echinatin (40 μM) or GA (20 μM), and then stimulated with nigericin, Lfn-FliC or poly(dA:dT). Cleaved caspase-1, production of IL-1β and cross-linked ASC in the Triton X-insoluble pellet were examined by IB analysis.

Data are expressed as mean ± SEM (n = 3/group, resulting from three independent experiments). One-way ANOVA followed by Dunnett's post-hoc test was used to assess the differences of multi-groups (B), $^{**}p < 0.01$, $^{***}p < 0.001$ compared to control.

Figure 5. Echinatin inhibits NLRP3 inflammasome activation in vivo and ameliorates LPS-induced septic shock

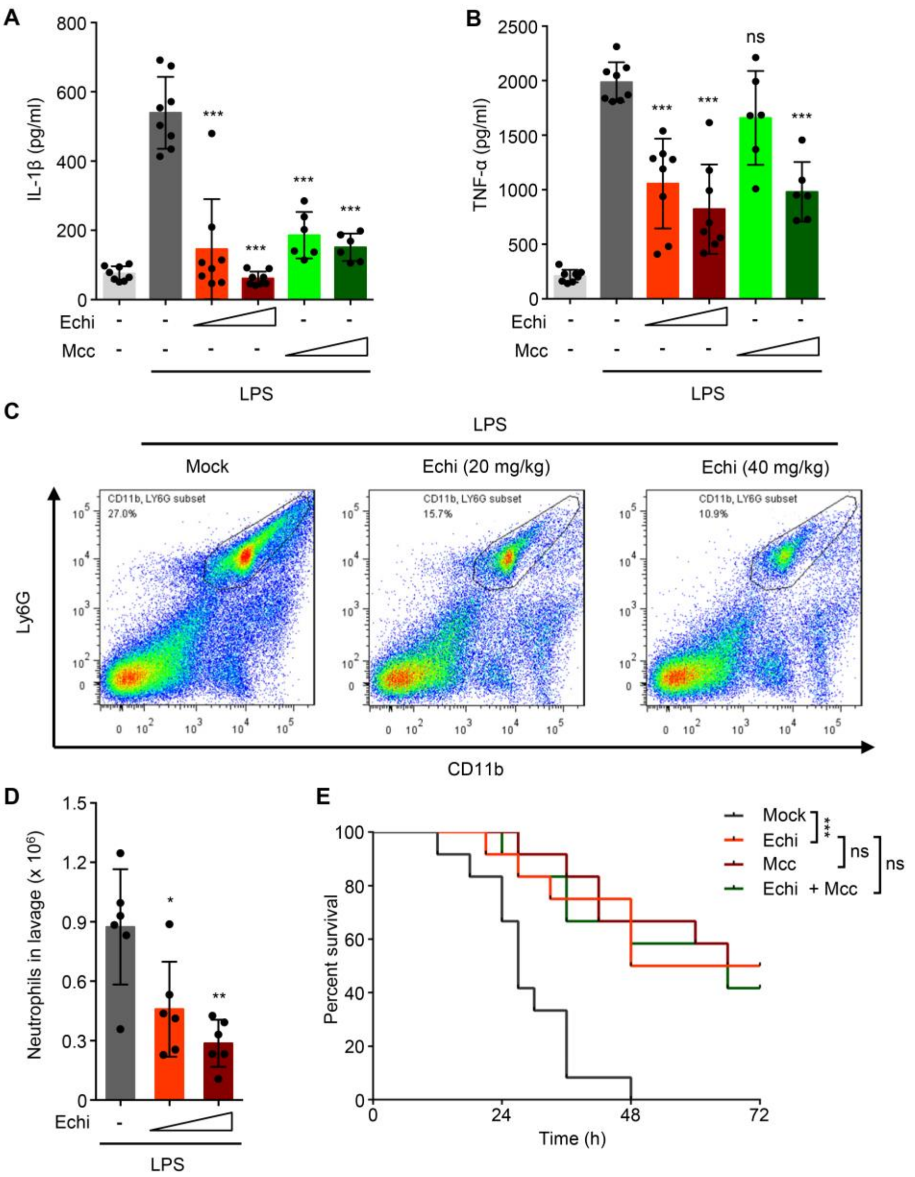

(A-B) ELISA of IL-1β (A) and TNF-α (B) in the serum of mice intraperitoneally injected with LPS (20 mg/kg body weight) in the presence or absence of echinatin (20 mg/kg and 40 mg/kg) and MCC950 (20 mg/kg and 40 mg/kg). (Mock-PBS, Mock-LPS, 20 mg/kg echinatin-LPS, 40 mg/kg echinatin-LPS, n = 8; 20 mg/kg MCC950-LPS, 40 mg/kg MCC950-LPS, n = 6).

(C) Representative FACS plots of neutrophils in the peritoneal cavity from mice intraperitoneally injected with LPS (20 mg/kg body weight) in the presence or absence of echinatin (20 mg/kg and 40 mg/kg).

(D) FACS analysis of neutrophil numbers in the peritoneal cavity described in (C). (n = 6 for each group).

(E) Survival of WT mice intraperitoneally injected with 20 mg/kg LPS that pretreated with vehicle (n = 12), echinatin (40 mg/kg, n = 12), MCC950 (40 mg/kg, n = 12), or the combination (n = 12).

Data are expressed as mean ± SD. One-way ANOVA followed by Dunnett's post-hoc test was used to assess the differences of multi-groups. ns: not significant, *p < 0.05, **p < 0.01, ***p < 0.001 compared to Mock-LPS (A, B, E) or to echinatin-LPS (D).

727 **Figure 6. Echinatin is efficacious in dextran sodium sulfate (DSS)-induced colitis model**

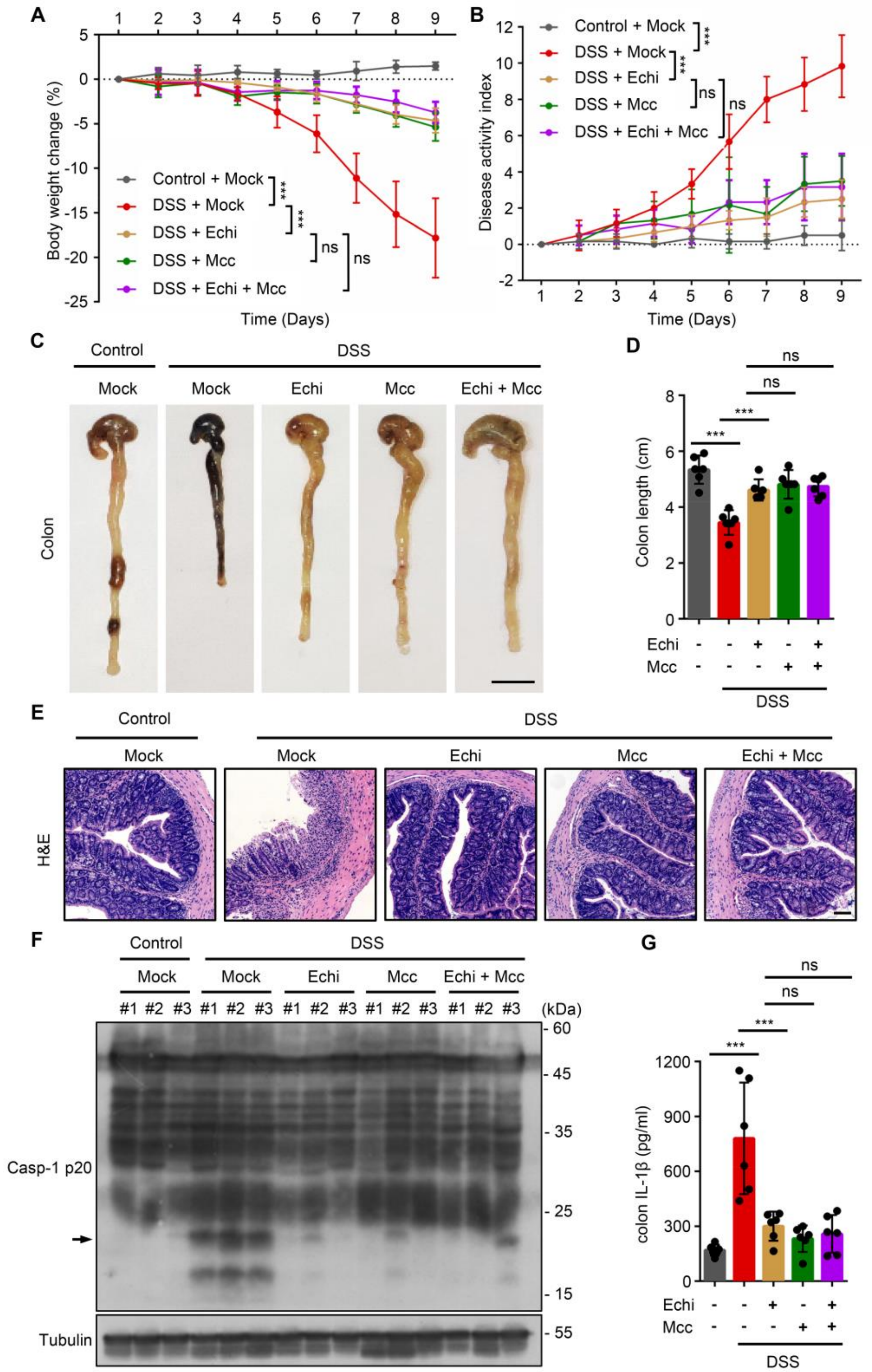

728

(A-B) WT C57BL/6 mice were given 2.5% DSS in the drinking water in the presence or absence of echinatin (40 mg/kg), MCC950 (40 mg/kg), or the combination for 9 days. Body weights (A) and disease activity index (B) of the mice were measured (n=6 for each group).

(C-E) Representative colon images (C), the colon lengths (D, n=6 for each group) and H&E-stained colon sections (E) were measured in 10th days after treatment with DSS plus vehicle, echinatin (40 mg/kg), MCC950 (40 mg/kg), or the combination. Scale bar (C), 1 cm. Scale bar (E), 200 μm.

(F-G) Representative IB analysis of active caspase-1 (F) and ELISA assay of IL-1β (G, n=6 for each group) in colon tissues.

Data are expressed as mean ± SD. One-way ANOVA followed by Least significant difference (LSD)'s post-hoc test was used to assess the differences of multi-groups (A, B, D, G). ***$p < 0.001$, ns: not significant.

Figure 7. Echinatin exhibits therapeutic effect in non-alcoholic steatohepatitis (NASH) model

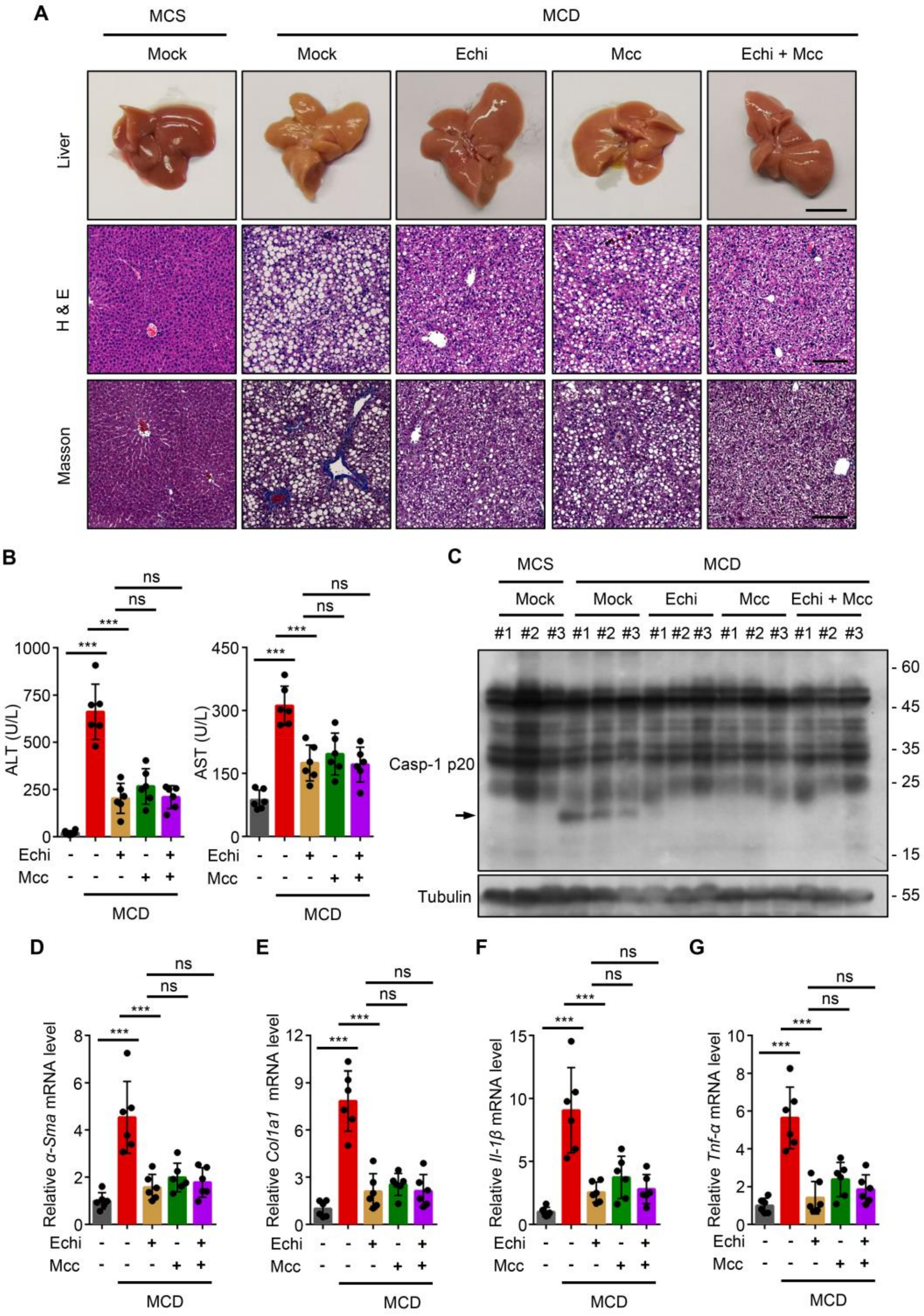

(A) Representative liver images, H&E-stained and Masson-stained liver sections are shown from the mice fed MCD or MCS diets in the presence or absence of Echi (40 mg/kg), MCC950 (40 mg/kg) or Echi plus MCC950. Scale bar (liver), 1 cm. Scale bar (H&E, Masson), 200 μm.

(B) The activity of plasma ALT and AST were measured described in (A). (n=6 for each group).

(C) Representative IB analysis of active caspase-1 level in liver tissues described in (A).

(D-G) Hepatic *α-Sma* (D), *Col1a1* (E), *Il-1β* (F) and *Tnf-α* (G) mRNA were measured from the mice described in (A). (n=6 for each group).

Data are expressed as mean ± SD. One-way ANOVA followed by Least significant difference (LSD)'s post-hoc test was used to assess the differences of multi-groups (B, D-G). ***p < 0.001, ns: not significant.

Supplementary Materials:

Figure S1. Echinatin had no effects on inflammasome-independent cytokine TNF-α production

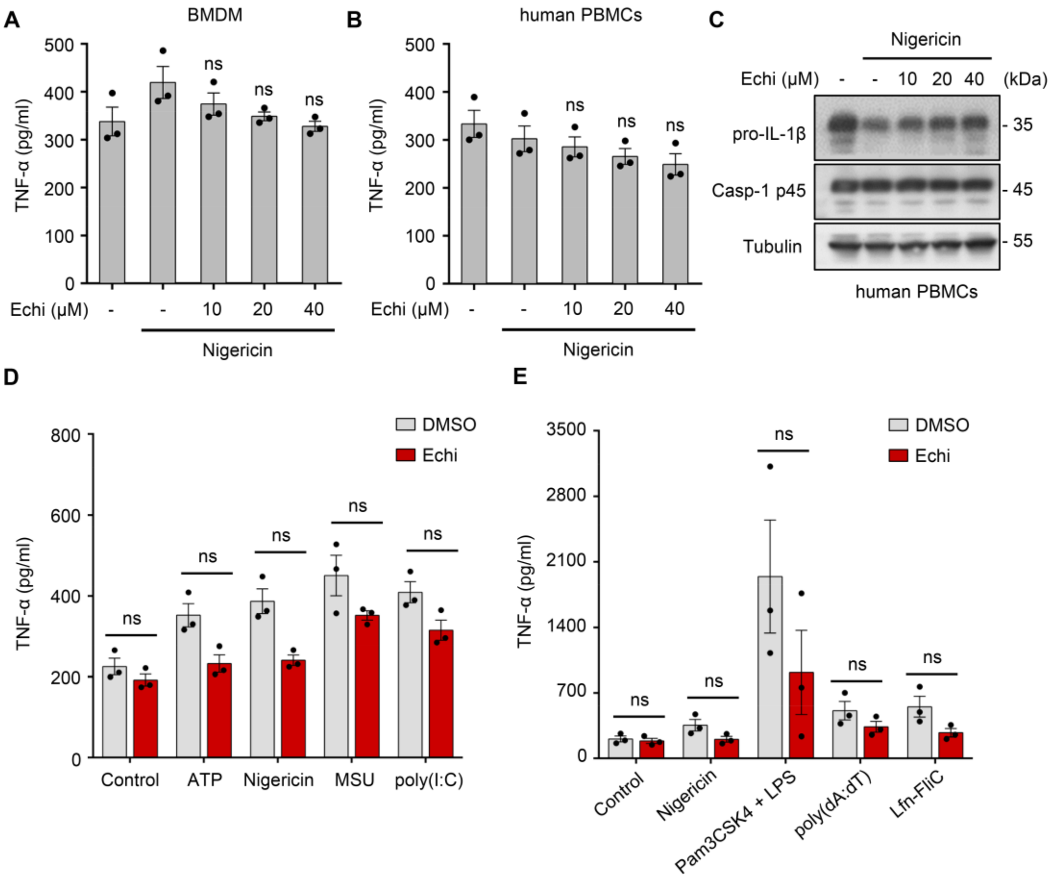

(A) Secretion of TNF-α were assessed in SN described in Figure 1C.

(B-C) Secretion of TNF-α in SN (B) and IB analysis of cell lysates (C) were assessed from the experiments described in Figure 1 G.

(D) Production of TNF-α in SN were assessed in SN described in Figure 2 A.

(E) Secretion of TNF-α in SN were assessed in SN described in Figure 3 A.

Data are expressed as mean ± SEM (n = 3/group, resulting from three independent experiments). Statistics differences were analyzed by one-way ANOVA followed by Dunnett's post-hoc test (A, B) or unpaired t test (D, E). ns: not significant.

Figure S2. Echinatin inhibits ATP-induced NLRP3 inflammasome activation and assembly in mouse BMDMs

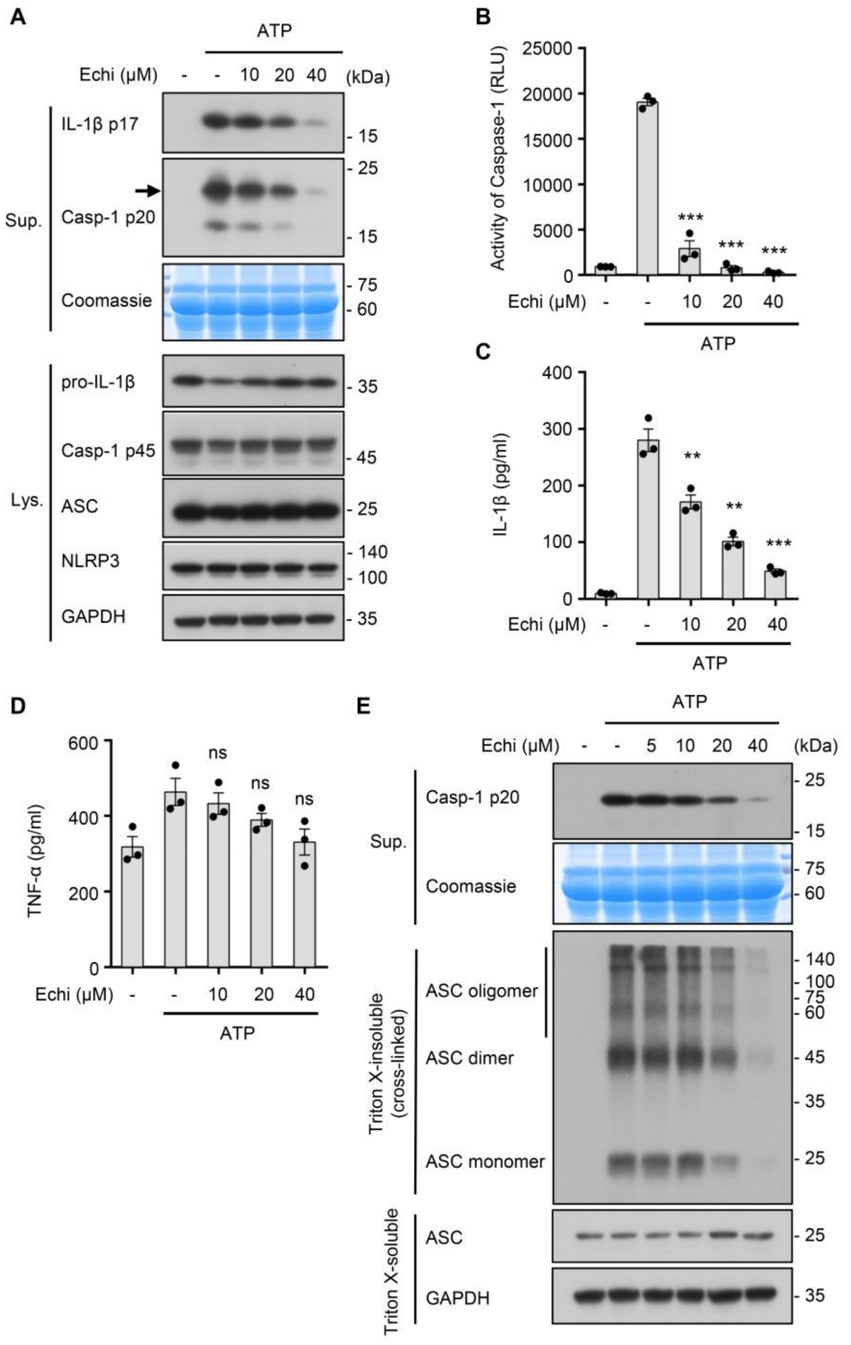

(A-D) LPS-primed BMDMs were pretreated with various doses of echinatin and then stimulated with ATP, cleaved caspase-1 and production of IL-1β were examined by IB analysis (A), activity of caspase-1 (B) and secretion of IL-1β (C), TNF-α (D) in SN were assessed.

(E) LPS-primed BMDMs were pretreated with indicated dose of echinatin and stimulated with ATP, IB analysis was used to detect cross-linked ASC in the Triton X-insoluble pellet.

Data are expressed as mean ± SEM (n = 3/group, resulting from three independent experiments). One-way ANOVA followed by Dunnett's post-hoc test was used to assess the differences of multi-groups (B-D). **$p < 0.01$, ***$p < 0.001$ compared to control, ns: not significant.

Figure S3. Echinatin is well tolerated and safe in mice

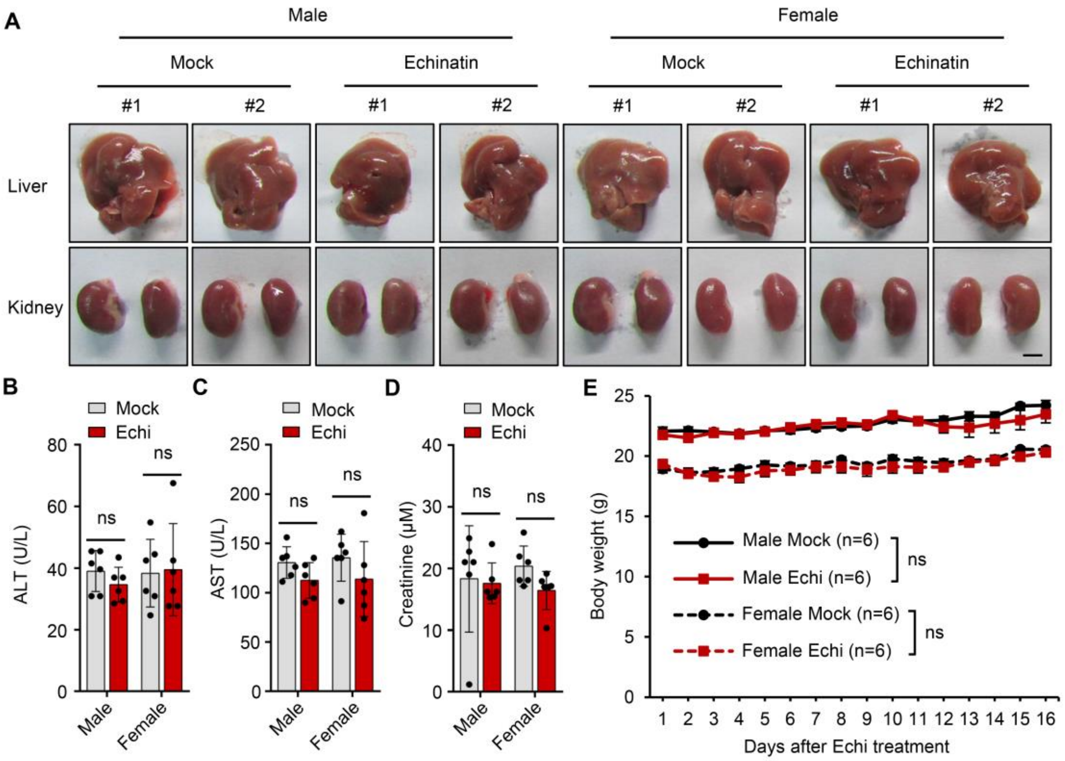

(A-E) Male and female C57BL/6 mice were treated with echinatin (120 mg/kg) daily for 15 days. Representative liver and kidney images (A), ALT (B) and AST (C) activity, Creatinine (D) level in the plasma were collected on day 16. Body weight (E) was measured every day. Scale bar: 0.5 cm.

Statistics differences were analyzed by one-way ANOVA followed by unpaired t test (B-E) (n = 6 for each group). ns: not significant.
